# A Review on Deep Learning for Quality of Life Assessment Through the Use of Wearable Data

**DOI:** 10.1109/OJEMB.2025.3526457

**Published:** 2025-01-14

**Authors:** Vasileios Skaramagkas, Ioannis Kyprakis, Georgia S. Karanasiou, Dimitris I. Fotiadis, Manolis Tsiknakis

**Affiliations:** Biomedical Informatics and eHealth LaboratoryDepartment of Electrical and Computer EngineeringHellenic Mediterranean University 112178 71410 Heraklion Greece; Institute of Computer ScienceFoundation for Research and Technology Hellas (FORTH) 54570 70013 Heraklion Greece; Biomedical Informatics and eHealth LaboratoryDepartment of Electrical and Computer EngineeringHellenic Mediterranean University 112178 71410 Heraklion Greece; Institute of Computer ScienceFoundation for Research and Technology Hellas (FORTH) 54570 70013 Heraklion Greece; Department of Science et TechniquesUniversity of Burgundy 27011 21000 Dijon France; Unit of Medical Technology Intelligent Information SystemsUniversity of Ioannina 37796 45110 Ioannina Greece; Biomedical Research InstituteFORTH 45110 Ioannina Greece

**Keywords:** Deep learning, healthcare, machine learning, quality of life, wearable data

## Abstract

Quality of Life (QoL) assessment has evolved over time, encompassing diverse aspects of human existence beyond just health. This paper presents a comprehensive review of the integration of Deep Learning (DL) techniques in QoL assessment, focusing on the analysis of wearable data. QoL, as defined by the World Health Organisation, encompasses physical, mental, and social well-being, making it a multifaceted concept. Traditional QoL assessment methods, often reliant on subjective reports or informal questioning, face challenges in quantification and standardization. To address these challenges, DL, a branch of machine learning inspired by the human brain, has emerged as a promising tool. DL models can analyze vast and complex datasets, including patient-reported outcomes, medical images, and physiological signals, enabling a deeper understanding of factors influencing an individual's QoL. Notably, wearable sensory devices have gained prominence, offering real-time data on vital signs and enabling remote healthcare monitoring. This review critically examines DL's role in QoL assessment through the use of wearable data, with particular emphasis on the subdomains of physical and psychological well-being. By synthesizing current research and identifying knowledge gaps, this review provides valuable insights for researchers, clinicians, and policymakers aiming to enhance QoL assessment with DL. Ultimately, the paper contributes to the adoption of advanced technologies to improve the well-being and QoL of individuals from diverse backgrounds.

## I. Introduction

The notion of Quality of Life (QoL) has been examined from multiple perspectives, resulting in the recognition that health-related QoL and total QoL are frequently synonymous. The World Health Organisation (WHO) characterises health as a holistic condition of physical, mental, and social well-being, underscoring its importance in improving quality of life. In addition to health, QoL includes employment capacity, social support, and the physical environment [1]. Researchers have suggested that QoL can be examined from several perspectives, such as psychological, economic, and medical, hence complicating its definition and assessment [2].

**Fig. 1.**
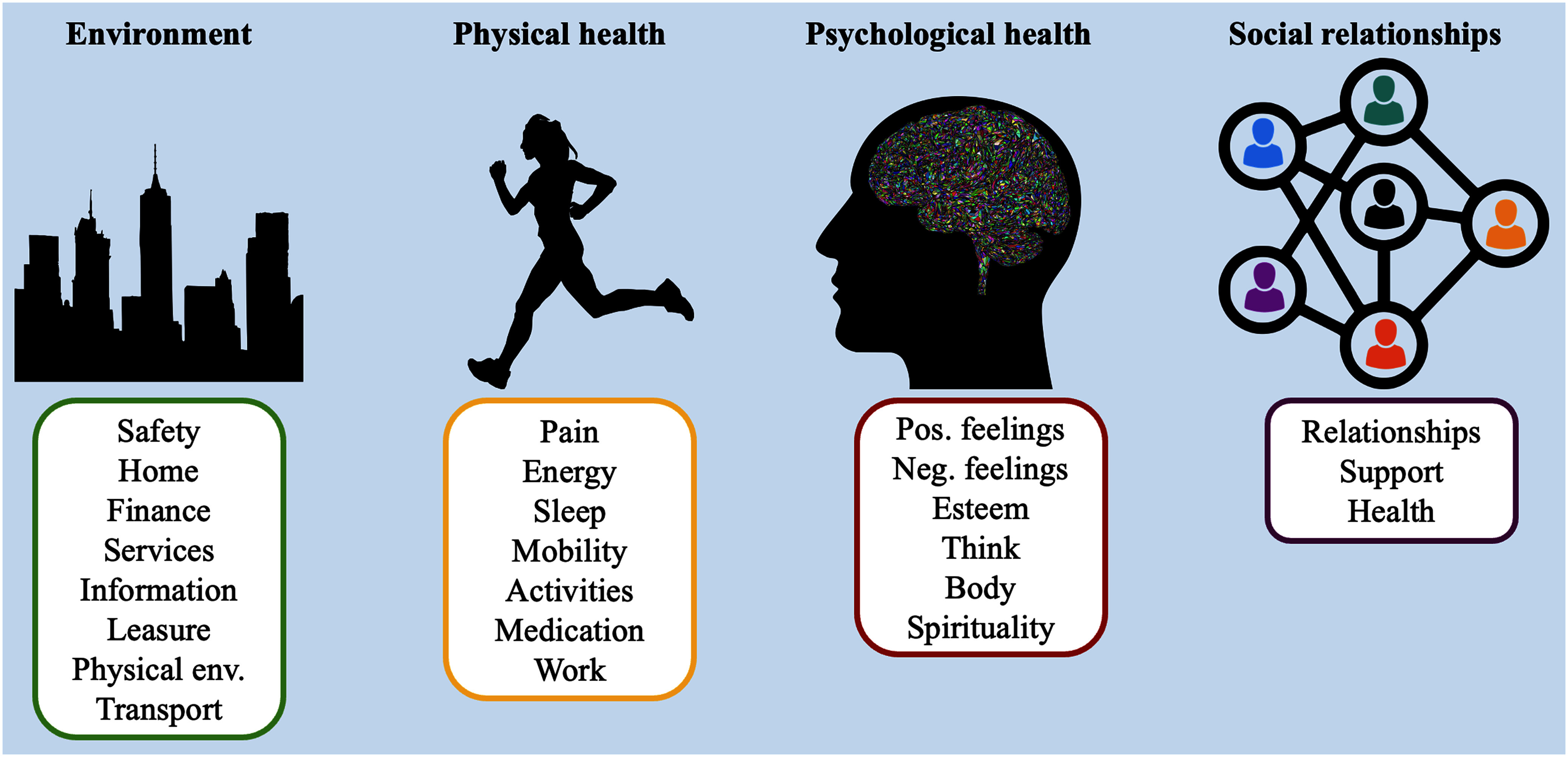
WHOQOL instrument domains and subdomains [5].

Conventional approaches to evaluating QoL have depended on informal enquiries by healthcare professionals, which may be subjective and variable. Two principal methodologies for systematic assessment have arisen: (1) validated patient-reported outcomes (PROs) instruments that gather subjective data [3]; and (2) objective data acquisition via technologies that record physiological signals and behaviours [3]. In response to the necessity for a thorough Quality of Life evaluation tool, the WHO created the WHOQOL assessment instrument, which includes many domains such as physical health, mental well-being, relationships, and environmental factors [4] (Fig. 1).

Recently, a paradigm change in QoL assessment has occurred with the incorporation of Deep Learning (DL) approaches, which utilise complicated datasets to improve comprehension of QoL domains [6]. This innovation facilitates the analysis of many data sources, such as PROs, medical imaging, and physiological signals, yielding enhanced insights into the determinants of quality of life [7]. Wearable technologies have significantly altered the landscape by providing continuous, real-time data on vital signs and other health parameters, thus improving the precision of quality of life assessments [8], [9]. This review examines the function of deep learning approaches in evaluating the physical and psychological health subdomains of QoL, emphasising the progress and prospective applications of wearable technology in this emerging and critical healthcare sector [10].

## II. Physical Health Assessment

The maintenance of physical health is an essential aspect that contributes significantly to an individual's holistic well-being. It comprises a broad spectrum of factors pertaining to the physiological functioning and overall welfare of the human body [11]. One of these factors, Human Activity Recognition (HAR), has progressed markedly due to the emergence of DL, utilising wearable sensor data to precisely categorise activities of daily living (ADL) such as walking, jogging, and driving. Convolutional Neural Networks (CNN) have exhibited remarkable efficacy in extracting spatial characteristics from sensor data, as evidenced by Dua et al. [12], where a CNN-GRU hybrid attained an accuracy of 96.00% across several datasets. Long Short-Term Memory (LSTM) networks, intended for sequential data, have proven effective, with Kuncan et al. [13] attaining 98.42% accuracy utilising Motif Patterns. Hybrid models such as CNN-LSTM [14] enhance performance, achieving accuracy levels of up to 99.00% on particular datasets. Recently, attention mechanisms and transformers have improved the accuracy of HAR, as demonstrated in Sarkar et al. [15] and Dirgova Luptakova et al. [16], where transformer-based models attained over 99.00% accuracy by effectively capturing temporal dependencies in sensor data. Nevertheless, numerous research, including those employing benchmark datasets like UCI-HAR, are constrained by restricted sample numbers and insufficient variety, which raises issues over their generalisability across populations with varying demographics or activity patterns. These constraints may impede the model's efficacy in varied real-world environments. Furthermore, datasets frequently inadequately represent the inherent diversity of human behaviours, leading to models that are tailored to certain, often idealised circumstances instead of the unpredictable nature of real-world situations.

Moreover, medication adherence, an essential element of effective therapy, has significantly improved with DL algorithms and wearable data, providing real-time feedback and accuracy in monitoring. Odhiambo et al. [17] used a Deep Neural Network (DNN) with accelerometer data from smartwatches to identify involuntary movements associated with medicine, attaining a precision of 96.50%. CNNs have been effectively utilised, as demonstrated by Lee et al. [18], who employed a camera image sensor combined with wearable devices to monitor medicine adherence, achieving an accuracy of 92.70%. CNN-based approaches for monitoring chronic diseases and glucose levels have shown encouraging outcomes [19]. Pettas et al. [20] employed LSTM networks, recognised for their capability in temporal data processing, to identify audio events from inhalers, achieving accuracy rates as high as 94.00%, surpassing conventional approaches.

Energy and fatigue (EF) are essential measures of an individual's health and productivity, with precise measurement vital for evaluating overall well-being. Recent studies have investigated innovative techniques for identifying EF using wearable sensors and deep learning models. Sharma et al. [21] employed CNN to monitor wrist motions and recognise eating events with an accuracy of 89.00%, whereas Wang et al. [22] integrated CNNs with attention mechanisms to assess eating speed, achieving a minimal error of 0.11. Advancements in mental fatigue detection have been made by deep learning approaches; Wu et al. [23] employed a Contractive Sparse Auto-encoder to categorise fatigue states from EEG data, attaining an accuracy of 83.00%. Bai et al. [24] utilised a self-attention LSTM model for fatigue detection using ECG and actigraphy data, illustrating the efficacy of integrating temporal and attention mechanisms. Additional significant applications involve employing CNNs and BiLSTM for the detection of driver sleepiness [25], [26] and utilising HRV signals from wearables to assess driver fatigue [27], with these models attaining accuracy rates of up to 94.31%. Notwithstanding these developments, a trade-off exists between the accuracy of high-performing models, such as hybrid CNN-LSTM architectures, and the feasibility of their implementation on resource-limited wearable devices. The computational requirements of these models, especially when managing extensive datasets or real-time data streams, may hinder their deployment on devices with limited processing capacity or battery longevity. This requires the investigation of more computationally efficient algorithms that can sustain high accuracy while remaining practical for wearable devices.

Mobility is another essential aspect of public health, encompassing physical mobility, ambulation, and transportation, all of which enhance an individual's QoL [28]. GPS-enabled wearables enable the assessment of life-space mobility, which is associated with social support and gait speed [29], whilst accelerometers monitor velocity and physical activity [30]. Numerous research efforts utilise wearable sensors to evaluate fall risk and mobility challenges, especially among the older population. Kulurkar et al. [31] attained a 95.87% accuracy in fall detection utilising LSTM and IoT-based systems. In patients with Parkinson's Disease, freezing of gait (FOG) was accurately predicted utilising transformer-based topologies combined with BiLSTM, resulting in elevated specificity and sensitivity [32].

Pain perception is a multifaceted and subjective experience that presents difficulties for objective assessment [33]. Recent breakthroughs in wearable technologies, including electrodermal activity (EDA) sensors and DL algorithms, provide novel methods for pain quantification, hence improving quality of life evaluations. Gkikas et al. [34] used multi-task learning (MTL) neural networks with ECG data, enhancing the precision of pain assessment. Rojas et al. [35] employed functional near-infrared spectroscopy and a BiLSTM model to attain 90.60% accuracy in evaluating pain in non-communicative patients. Pouromran et al. [36] enhanced pain intensity classification using a customised BiLSTM model, achieving a f1-score of 0.81 and an AUROC of 0.93 across multiple pain states. Hu et al. [37] proved the efficacy of LSTM in chronic pain identification, attaining precision and recall rates of 97.20% via balance and body sway analysis. Wang et al. [38] investigated protective behaviour recognition with layered LSTM methodologies, achieving an ideal F1-score of 0.82. Furthermore, Yu et al. [39] employed EEG signals to objectively assess pain, attaining classification accuracy of 97.37%, via CNN-based models.

Additionally, sleep is a vital physiological condition marked by a transient loss of consciousness and modified cerebral activity, serving a fundamental function in both physical and mental well-being [40]. Emerging wearable technology and sophisticated deep learning approaches are crucial for precisely measuring sleep, improving personal understanding of sleep patterns, and aiding healthcare professionals in detecting sleep problems and refining treatment strategies. The NetHealth dataset [41], which examined data from 698 college students, revealed that CNN could proficiently evaluate sleep quality, attaining a mean absolute error (MAE) of roughly 0.04. Furthermore, Yildirim et al. [42] presented a 1D-CNN model that automated the classification of sleep stages utilising polysomnogram (PSG) data, attaining accuracies ranging from 91.00% to 98.06%. Mousavi et al. [43] created SleepEEGNet, which employed single-channel EEG data to attain an accuracy of 84.26% by integrating CNN and sequence-to-sequence models. In contrast, Supratak et al. [44] merged CNN and BiLSTM networks in the DeepSleepNet model, achieving an accuracy of 86.20%. Furthermore, actigraphy sensors have shown efficacy in forecasting sleep efficiency, with CNN achieving the best accuracy of 97.30% [45]. LSTM models, as emphasised by Phan et al. [46], successfully forecasted sleep quality via physical activity data, attaining an accuracy of 61.00. Finally, Matsumori et al. [47] utilised a hybrid CNN-LSTM model with a lightweight EEG sensor, attaining an accuracy of 78.60%, equivalent to clinical PSG systems.

Lastly, work capacity, as defined by the American College of Sports Medicine (ACSM), refers to the maximum physical work an individual can perform, assessed through power output or endurance and influenced by factors like cardiorespiratory fitness and muscular strength [48]. Traditional evaluations have relied heavily on self-report instruments, which often suffer from reliability issues due to biases and recall problems [49]. The Work-ability Support Scale (WSS) effectively measures vocational capability after disability, covering physical, cognitive, and social domains [50]. Other assessments, such as the Functional Capacity Evaluation (FCE) and the Work Ability Index (WAI), focus on job-specific physical and cognitive requirements [51]. The Work Capacity Test (WCT), used by organizations like the U.S. Forest Service, assesses physical capabilities for demanding roles [52]. Wearable activity trackers can quantify many work capacity factors, making them useful for physically demanding jobs. However, like mobility, we assume that the subset of work capacity that can be evaluated using DL using wearable sensor data is closely connected with ADL evaluation.

For a comprehensive summary of studies employing wearable devices for physical health assessment, including datasets, sensors, and methodologies, we refer readers to Table I in the supplementary material.

## III. Psychological Health

The QoL of an individual is significantly influenced by their physiological health, encompassing various dimensions such as feelings, self-esteem, memory, spirituality, and body image [53]. The importance of physiological health within the larger framework of QoL becomes apparent when we consider its direct influence on many domains. One such domain, feelings, encompassing both positive and negative states, are fundamental to well-being and QoL [54]. Recent advancements in wearable technology have significantly improved the ability to identify emotions by monitoring physiological signals such as heart rate variability, skin conductance, and facial expressions [55]. Research employing DL methodologies has demonstrated this potential; for instance, the eSEE-d database utilises eye-tracking data for emotion estimation, achieving an accuracy of up to 92.00% for positive valence [56]. Furthermore, systems integrating sensors with deep learning models, such as a smartwatch-based adaptive system for multi-sensory emotion detection, have attained an accuracy of 74.30% in identifying arousal and valence [57]. In addition, self-supervised learning has shown robustness to data degradation, achieving 81.00% accuracy in emotion recognition [58], while emotion recognition in older adults using LSTM networks has reached accuracies of up to 95.00% [59]. Moreover, hybrid CNN-LSTM models have demonstrated efficacy with precision rates as high as 99.00% [60]. Large Language Models (LLMs) like GPT have been utilized for analyzing patient narratives and emotion estimation, complementing sensor-based methods for psychological health assessment. For example, recent studies [61] have demonstrated how these models can process unstructured text data to derive insights into emotional well-being, thereby enriching the understanding of QoL dimensions.

Self-esteem, which refers to an individual's self-acceptance and self-regard, is shaped by personal and cultural standards and their perceived competency in essential life domains [62]. Traditionally, self-esteem evaluations have relied on self-report instruments such as the Rosenberg Self-Esteem Scale (RSE) and the Single Item Self-Esteem Scale (SISE) [63], [64]. Instruments like the Multidimensional Self-Esteem Inventory (MSEI) and the Contingency of Self-Worth Scale (CSWs) focus on specific dimensions of self-esteem [65], [66]. However, wearable technology presents innovative yet complex possibilities for measuring self-esteem. A novel method utilising EEG data and CNN models has achieved an accuracy exceeding 79.00% in differentiating between high and low self-esteem [67]. Although CNN-LSTM models demonstrate great accuracy in emotion recognition, their lack of explainability hinders healthcare practitioners from trusting and implementing these methods in practice. The opaque nature of deep learning models hinders the interpretability of outcomes, particularly in sensitive domains like psychological health, where practitioners require clear and comprehensible insights for informed decision-making. Explainable AI (XAI) models are necessary to overcome these concerns and enhance trust in such technologies for clinical application.

Spirituality, which encompasses the acknowledgment of a higher force and the pursuit of meaning beyond sensory experience, poses unique challenges for technological quantification [68]. Instruments such as the Spiritual Well-Being Scale (SWBS) [69], the Spiritual Needs Questionnaire (SpNQ) [70], and the Spirituality Questionnaire [71] are commonly employed to evaluate spiritual well-being. Despite the promise offered by wearable sensors and deep learning for quality of life assessments, the subjective and contextual nature of spirituality presents considerable obstacles, as physical data may inadequately represent spiritual experiences.

Thinking, comprising fundamental mental processes such as perception, memory, problem-solving, and decision-making, is vital for numerous aspects of life, including emotional control and communication. Recent advancements in DL have facilitated the classification of cognitive states through wearable devices. For example, integrating EEG data with CNN models has achieved an accuracy of up to 96.70% in classifying cognitive workload in drivers [72]. Similarly, DL approaches employing EEG and eye-tracking data have shown great accuracy (up to 97.00%) in identifying cognitive effort and mental burden [73]. These methodologies, despite facing obstacles, exhibit great potential for enhancing cognitive evaluation and, consequently, QoL.

Body image refers to an individual's cognitive and emotional perceptions regarding their physique, encompassing elements such as form, size, and attractiveness [74]. While wearable devices like smartwatches and activity trackers can gather data on physical metrics such as blood pressure and bodily movements [75], they are limited in their ability to encapsulate the intricate, subjective aspects of body image, including body acceptance and self-perception [76]. A comprehensive evaluation of body image necessitates an amalgamation of objective metrics and self-reported instruments, including the Body-Image Acceptance and Action Questionnaire [77] and the Body Image Scale [78]. By integrating these diverse elements, we can gain a more nuanced understanding of psychological health and its impact on overall quality of life.

For a comprehensive summary of studies employing wearable devices for psychological health assessment, we refer readers to Table II in the supplementary material.

## IV. Publicly Available Datasets

This section discusses the strengths and weaknesses of datasets related to QoL subdomains that include wearable sensor data and are publicly accessible. Table I highlights significant variation in participant data, with sample sizes ranging from 4 (“OPPORTUNITY”) to 700 (“NetHealth”) and ages spanning 18 to 78 years, as seen in the “Sleep-EDF” dataset. Such diversity enhances the generalizability of findings across age cohorts. Gender distribution also varies; for instance, “BioVid Heat Pain” includes 43 females and 44 males, while “MHEALTH” lacks gender-specific data. Demographic diversity aids in understanding how factors like age and gender influence QoL assessments through wearable data [105].

**TABLE I:** Summary of Identified Publicly Available Datasets Containing Wearable Data and Stimuli Related to QoL Domains

**Dataset**	**Subjects**	**Age**	**Gender (F/M**)	**Stimuli**	**Wearable data**	**Subdomain**
UCI-HAR [79]	30	19-48		6 ADL activities	ACC, GYRO (50 Hz)	ADL
WISDM [80]	36			6 ADL activities	ACC (20 Hz)	ADL
PAMAP [81]	9	27.2$\pm$ 3.3	1/8	18 ADL activites	ACC, GYRO, HR (100 Hz)	ADL
Extra-sensory [82]	60	18-42	34/26	51 behavioural activities	ACC, GYRO, MAG (40 Hz), Watch ACC (25 Hz), GPS, Audio	ADL
OPPORTUNITY [83]	4			5 ADL morning activities	ACC, GYRO, MAG (30 Hz)	ADL
UniMib-SHAR [84]	30	18-60	6/24	9 ADL activities, 8 falls	ACC (50 Hz)	ADL
Daily and Sport Activities [85]	8	20-30	4/4	19 ADL and sports activities	ACC, GYRO, MAG (25 Hz)	ADL
REALWORLD16 [86]	15	31.9$\pm$ 12.4	8/7	6 ADL activities	ACC, GYRO, MAG, Location, Audio	ADL
MHEALTH [87]	16			12 physical activities	ACC, GYRO, HR, ECG (50 Hz)	ADL
BioVid Heat Pain [88]	87	20-65	43/44	Heat stimulus	ECG, EMG, SCL	Pain
EmoPain [89]	50	44 (mean)	29/21	Physiotherapy activities	ACC, GYRO, EMG (1 kHz)	Pain
MobiAct [90]	57	20-47	15/42	Falls	ACC, GYRO (20 Hz)	Mobility
MIT/BIH PSG [91]	16	32-56	0/16	Whole-night sleep recordings	EEG, EOG, EMG, BVP, OS, RS, CV (250 Hz)	EF
FD I&II [92]	61			Whole-day eating episodes	IMU (64 Hz)	EF
Sleep-EDF [93]	22	18-78	7/15	Whole-night sleep recordings	EEG, EOG, EMG (50 Hz)	EF/Sleep
NetHealth [94]	700			ADL activities, sleeping task	HR, Sleep biomarkers	Sleep
Sleep-EDFX [95]	24	18-79	15/9	Whole-night sleep recordings	EEG, EOG, EMG (50 Hz)	Sleep
MASS [96]	200	18-76	103/97	Whole-night sleep recordings	EEG, EOG, EMG, ECG, RS (256 Hz)	Sleep
Apnea ECG [97]	27	27-63	6/21	Whole-night sleep recordings	ECG (100 Hz)	Sleep
eSEE-d [56]	48	18-47	27/21	Emotion evoking videos	Eye tracking metrics	Feelings
AffectiveROAD [98]	10	24-34	5/5	Real world driving sessions	BVP, ACC (36 Hz), EDA (4 Hz), HR (1 Hz), ECG, BR, ST (4 Hz)	Feelings
CASE [99]	30	22-37	15/15	Emotion evoking videos	ECG, BVP, EMG, EDA (1000 Hz)	Feelings
CLAS [100]	60	20-50		Emotion evoking videos, mentally demanding tasks	ECG, PPG, EDA, ACC (256 Hz)	Feelings
K-EmoCon [101]	32	19-36	12/20	Naturalistic conversations	ECG (1 Hz), EEG (125 Hz), BVP (64 Hz), EDA (4 Hz), BT (4 Hz), ACC (32 Hz), HR (1 Hz)	Feelings
PPG-DaLiA [102]	24	26.9$\pm$ 4.8	14/10	Walking activities	PPG, ECG, ACC, GYRO	Feelings
WESAD [103]	15	24-35	3/12	Sedentary activities	BVP (64 Hz), ACC (32 Hz), EDA (700 Hz), BT (700 Hz),EMG (700 Hz), BR, ECG (700 Hz)	Feelings
DEAP [104]	32	19-37	16/16	Music videos	EEG (512 Hz)	Feelings

ACC: Accelerometer, GYRO: Gyroscope, HR: Heart Rate, MAG: Magnetometer, GPS: Global Positioning System, EEG: Electroencephalogram, EOG: Electrooculogram, EMG: Electromyogram, BVP: Blood Volume Pulse, OS: Oxygen Saturation, RS: Respiration, CV: Cardiovascular, ECG: Electrocardiogram, SCL: Skin Conductance Level, EDA: Electrodermal Activity, PSG: Polysomnography, BR: Breathing Rate, ST: Skin Temperature, BT: Body Temperature, PPG: Photoplethysmogram, UV: Ultraviolet radiation.

The datasets encompass a wide array of stimuli and activities, demonstrating the adaptability of wearable technology in evaluating various facets of daily life. For instance, “Extra-sensory” assesses 51 behavioral activities, whereas “MIT/BIH PSG” concentrates on overnight sleep recordings. Numerous datasets, like “UCI-HAR,” “WISDM,” and “PAMAP,” focus on ADL, rendering them especially pertinent for quality of life evaluations in this subdomain. In contrast, datasets such as “MIT/BIH PSG” and “Sleep-EDF” focus on sleep-related stimuli, corresponding to the Energy and Fatigue, and Sleep (EF/Sleep) subdomains. This variability enables researchers to customize their inquiries to particular aspects of QoL. Furthermore, the datasets employ several wearable sensors, such as accelerometers (ACC), gyroscopes (GYRO), and electrocardiograms (ECG), to assess quality of life (QoL) thoroughly. The MHealth dataset integrates ACC, GYRO, heart rate (HR), and ECG data, rendering it suitable for assessing activities of daily living (ADL) in QoL research. Likewise, EEG and EMG data in “Sleep-EDFX” and “MASS” are customized for sleep-related subdomains. The emotional aspects of quality of life are examined in datasets such as “eSEE-d” and “CASE,” which utilize emotion-inducing films and record physiological signals like ECG and electrodermal activity (EDA). The diversity and richness of wearable data in these datasets provide a detailed examination of quality of life across many research requirements.

## V. Conclusion

In conclusion, integrating DL with wearable technology offers a promising approach to evaluating QoL, excelling in domains like physical and psychological well-being. Models like CNN and LSTM provide accurate insights into daily activities, medication adherence, and mental states through real-time, objective data often missed by self-reports. DL's ability to process multimodal sensor data enables comprehensive, dynamic, and personalized QoL assessments. However, challenges remain regarding generalizability, data variability, and privacy. Limited datasets and demographic-specific studies hinder broader applicability, while subjective aspects like body image and spirituality pose integration difficulties. Real-world deployment faces hurdles like noisy data, battery constraints, and privacy concerns.

Looking ahead, innovations like explainable AI, federated learning, and edge computing promise more transparent, private, and real-time wearable data processing. Interdisciplinary collaboration is essential for advancing DL-driven QoL evaluations, paving the way for transformative impacts on healthcare and well-being.

## Conflict of Interest

The authors declare no conflict of interest.

## Supplementary Materials

Supplementary Materials
