# Supplementary material for: A Review on Deep Learning for Quality of Life Assessment Through the Use of Wearable Data
Source: IEEE Open J Eng Med Biol. 2025 Jan 14;6:261–8. doi: 10.1109/OJEMB.2025.3526457 (PMC11793860; doi:10.1109/OJEMB.2025.3526457)
Supplement: Supplementary Materials [file supp1-3526457.pdf]

Supplementary Materials

A Review on Deep Learning for Quality of Life Assessment Through the Use of Wearable Data

Vasileios Skaramagkas, Ioannis Kyprakis, Georgia S. Karanasiou, Dimitris I. Fotiadis, *Fellow, IEEE*, **and Manolis Tsiknakis,** *Member, IEEE*

I. Revolutionizing Quality of Life Assessment: Integrating Deep Learning and Wearable Technologies

A MULTITUDE of approaches exist to conceptualise the notion of Quality of Life (QoL). Over the years, certain scholars have proposed that the definitions of health-related QoL and QoL can be employed interchangeably, indicating that the role of health in enhancing QoL is significant. According to the World Health Organisation (WHO), health is characterised as a state of comprehensive physical, mental, and social well-being, rather than simply the absence of sickness or infirmity [1]. Nevertheless, it is important to note that QoL encompasses other crucial aspects except just health, such as employment capacity, social support, and physical environment [2]. In essence, the concept of QoL can be understood from various perspectives, including psychological, economic, policy, and medical science viewpoints [3]. It can also be analysed by categorising it into individual aspects such as physical or psychological well-being.

Taking into consideration all of the aforementioned factors, it becomes evident that there is a divergence of perspectives regarding the precise definition of QoL. Consequently, this divergence poses significant challenges in the process of quantifying and -as a result- measuring the QoL. The measurement of an individual's QoL enables a comprehensive evaluation of their condition across various domains and can provide valuable insights for therapeutic decision-making. Specifically, QoL assessment and evaluation is crucial for patient-centered care, enabling tailored treatments, measuring treatment effectiveness, optimizing resource allocation, upholding ethical principles, aiding in long-term care planning, fostering research and innovation, informing health policy, offering psychosocial support, ensuring legal and regulatory compliance, and ultimately enhancing overall healthcare outcomes by prioritizing patients' well-being and preferences [4], [5].

Throughout the course of time, QoL assessment has been conducted indirectly by doctors or physicians commonly inquiring about the patient's condition through informal questioning, such as asking about their current well-being or the status of their symptoms. Nevertheless, in order to consistently evaluate the QoL in clinical decision making, two primary methods are employed: (1) using validated patient-reported outcomes (PRO) instruments to collect subjective self-reported data on different aspects of individuals' lives [6], [7]; and (2) utilising technologies to objectively gather biological samples, physiological signals, behaviours, or interactions with the environment [7], [8].

In order to address the necessity of developing a comprehensive evaluation of QoL that can be effectively used in many cultural contexts, the World Health Organisation Quality of Life (WHOQOL) assessment instrument was created by the WHOQOL Group in partnership with fifteen international field centres. As per the WHOQOL assessment [10], QoL is described as ”an individual's subjective evaluation of their status in life, considering the cultural and value systems of their environment, as well as their personal goals, expectations, standards, and concerns.” The WHOQOLBREF tool is employed to assess an individual's quality of life in four primary domains: physical health, mental well-being, relationships, and environmental influences [9]. The domains are separated into 24 subdomains, which are referred to as 'facets' [10] (Fig. 1). The subdomains encompass both subjective and objective dimensions of human existence, and they are not mutually exclusive but rather have the ability to interweave.

In recent years, the field of QoL assessment has witnessed a paradigm shift with the integration of Deep Learning (DL) techniques. DL, which is a branch of machine learning (ML) that draws inspiration from the anatomical and functional characteristics of the human brain, has demonstrated considerable potential in deriving significant and valuable knowledge from intricate and unorganised datasets [11], [12]. The utilisation of this technology possesses the capability to significantly transform the assessment of QoL domains by facilitating the analysis of extensive datasets that contain a wide range of characteristics pertaining to people's lives [13], [14]. Within the realm of QoL evaluation, DL models have the potential to be utilised for the automated analysis and interpretation of many sources of information. These sources include patient-reported outcomes, medical images, physiological signals, and even interactions on social media platforms [15]–[17] and can be related to wide spectrum of diseases such as cardiovascular [18] or Parkinson's [19]. Furthermore, foundation models and large language models (LLMs), such as GPT and BERT, have shown promise in complementing wearable data analysis by processing unstructured textual inputs like patient-reported outcomes and social media interactions, offering deeper contextual understanding of QoL parameters [20]. Acknowledging the aforementioned PRO instruments as the 'Ground Truth' against which DL algorithms can be validated, then, by harnessing the power of Artificial Intelligence (AI), researchers and healthcare practitioners can gain a deeper understanding of the factors influencing an individual's QoL, leading to more personalized and effective interventions.

Furthermore, significant progress has been made in the realm of research concerning the development of wearable

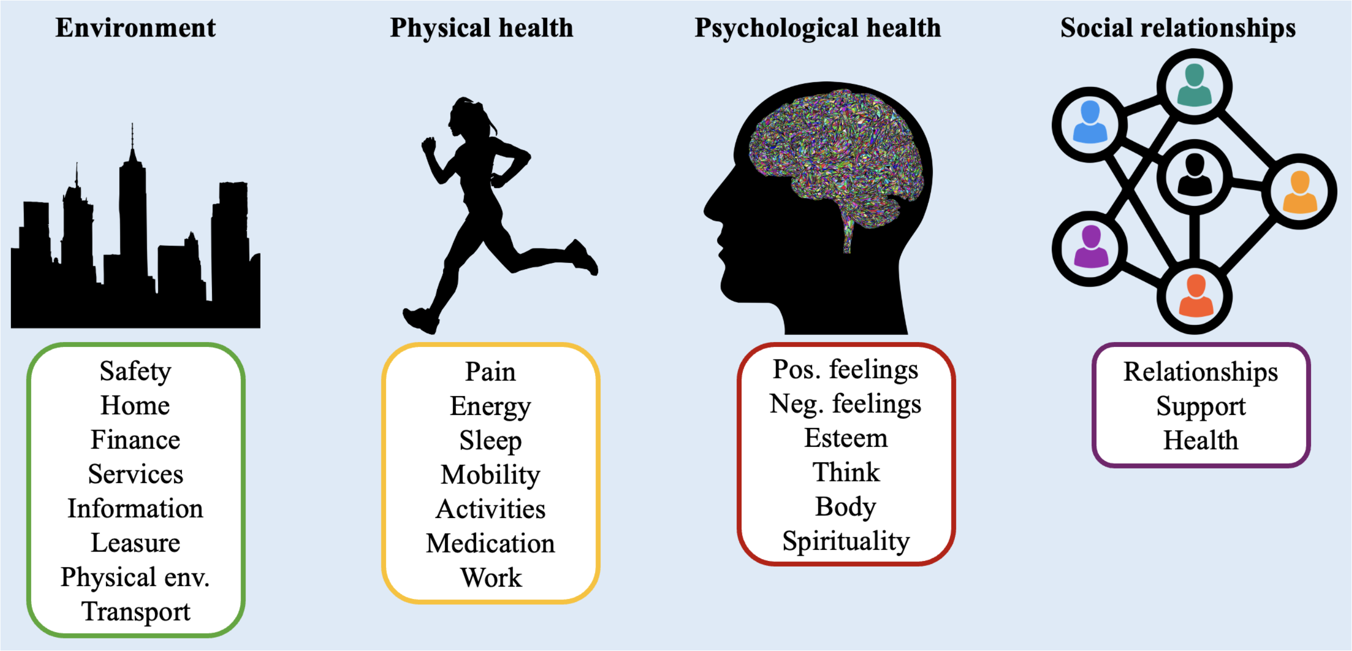

Fig. 1. WHOQOL Instrument Domains and Subdomains [9].

sensory devices. These devices possess the potential to be placed in different anatomical locations and are capable of connecting to a range of modalities, both individually and in combination. The effective exploitation of sensors and the meticulous selection of the analysed modalities may enable the autonomous and unobtrusive capture of real-world data [21]. In the realm of healthcare, wearable technology has the potential to provide immediate and continuous data on a patient's vital signs [22], [23]. This enables professionals to swiftly discover key aspects that define the overall QoL [24]. This advantage is valid irrespective of whether the assessment is conducted in a clinical environment or by remote means. Therefore, the usage of sensors for the acquisition of biomarkers from different modalities assumes great importance, as it facilitates the provision of real-time monitoring capabilities.

In this review we focus on assessing the subdomains of physical and psychological health while omitting the evaluation of social relationships and environmental factors [21]. This selective approach is rooted in the inherent limitations of data collection through wearable devices. Wearable technology primarily captures physiological and behavioral data related to an individual's physical and mental well-being. Parameters such as heart rate, activity levels, sleep patterns, and stress indicators can be effectively monitored using these devices, making them well-suited for assessing physical and psychological health [21]. However, when it comes to evaluating social relationships and environmental factors, the scope of wearable data is more restricted [25]. Assessing complex aspects of social interactions and the multifaceted nature of environmental influences requires data sources and methodologies beyond the capabilities of wearable devices [26]. Thus, this deliberate focus on physical and psychological health is not only pragmatic but also aligns with the potential of wearable technology to offer meaningful insights into these domains, contributing significantly to the overall goal of comprehensive QoL assessment.

The primary objective of this comprehensive review is to conduct a complete analysis of the use of DL techniques in the assessment of QoL and its domains. We will delve into the existing literature, highlighting the key advancements, challenges, and opportunities in this emerging field. Our review aims to provide a comprehensive evaluation of the several DL methodologies employed in directly assessing elements of QoL by identifying key papers to capture a broad spectrum of DL applications in wearable data for QoL assessments across various physical and psychological health tasks. This will encompass an examination of predictive model construction, the extraction of features from multimodal data, and the interpretation of model outputs. This study seeks to offer significant insights to academics, clinicians, and policymakers who are interested in utilising DL for the purpose of improving the assessment of QoL. Ultimately, our contribution lies in facilitating the adoption of advanced technologies to improve the well-being and QoL for individuals across diverse cultural and socio-economic backgrounds.

Our research has its roots in the needs and objectives of the CARDIOCARE project, reflecting our dedication to improving healthcare outcomes and enhancing QoL [27]. This project, supported by the Horizon 2020, aims to tackle the intricate issues encountered by elderly breast cancer patients, with a specific emphasis on therapy-induced cardiac toxicity. CARDIOCARE promises to empower patients by employing a comprehensive strategy involving eHealth applications, wearable sensors, and biomarkers to improve physical and mental health and create personalised care plans [28]. CARDIOCARE aims to reduce negative occurrences, hospitalisations, and healthcare expenses by using successful risk assessment and prevention methods. This immediately improves the QoL for this at-risk

group of patients.

II. Physical Health Assessment

The maintenance of physical health is an essential aspect that contributes significantly to an individual's holistic well-being. It comprises a broad spectrum of factors pertaining to the physiological functioning and overall welfare of the human body [29]. The evaluation of an individual's physical well-being is of utmost importance in comprehending their capacity to engage in everyday tasks, effectively manage health ailments, and attain a sense of contentment and fulfilment in life. The progress made in wearable technology has created opportunities for the real-time evaluation and tracking of various aspects of physical health. This literature review identified a number of DL methodologies in utilising wearable data for the evaluation of physical health and its facets. It highlights the significant influence of technology on our comprehension and control of physical well-being. The summary of the identified studies is presented in Table 1.

A. Activities of Daily Living (ADL)

The sector of Human Activity Recognition (HAR), which relies on data collected from wearable devices, has witnessed significant expansion, primarily propelled by improvements in machine learning methodologies. Several methods based on DL have been utilised to discern physical exercises and other activities conducted by individuals in their daily routines [79]. Activities such as jogging, walking, climbing stairs, washing dishes, and driving can be reliably identified. A number of notable architectural designs have surfaced in the field, such as Convolutional Neural Networks (CNN), Long Short-Term Memory (LSTM) as well as attention mechanisms.

CNN have proven to be highly efficient in HAR because of their capacity to capture spatial hierarchies in data using convolutional layers. CNN perform exceptionally well in tasks that require the extraction of spatial features from unprocessed sensor input. In the study conducted by Dua et al. [30], a CNN integrated with a Gated Recurrent Unit (GRU) shown exceptional accuracy rates on various datasets. Specifically, it achieved a 96.20% accuracy on the UCI-HAR dataset, 97.21% on the WISDM dataset, and 95.27% on the PAMAP dataset. The utilisation of CNN's feature extraction skills in conjunction with GRU's proficiency in processing sequential data showcased a superior performance compared to conventional handcrafted feature extraction techniques. Recently, a deep graph CNN within their HAR-GCCN framework was employed in Mohamed et al. [32] that leverages the correlation between chronologically adjacent sensor measurements to predict the correct labels for unclassified activities that have at least one activity label. The aforementioned methodology exhibited a substantial enhancement in the accuracy of classification, surpassing the performance of standard techniques by a margin of 68.00% across diverse datasets.

In a layer-wise manner, the effectiveness of CNN with local loss in the domain of HAR has been demonstrated by across several datasets in [33]. The study shown that local loss exhibited superior performance compared to global loss, even when utilising a lesser parameter count. This finding emphasises the potential of local loss in enhancing the effectiveness of current deep HAR methodologies. Furthermore, in their study, scientists in [34] developed a system utilising deep CNN to detect and classify driving behaviours. The study demonstrated the effectiveness of utilising AlexNet in accurately recognising distracted driving behaviours, with an average accuracy rate of 81.60%. This research highlighted the benefits of employing DL techniques in this context.

LSTM networks, which are specifically built to capture long-term dependencies in sequential data, have also been extensively utilised in the field of HAR. The research conducted by Kuncan et al. [35] employed Motif Patterns (MP) to extract features and utilised LSTM for classification, resulting in a 98.42% success rate. The LSTM's capacity to preserve information across extended sequences renders it well-suited for tasks that require the analysis of temporal patterns. An additional example is the RLSTM model introduced by Mekruksavanich et al. [38], which improved upon the fundamental LSTM architecture to attain a remarkable accuracy of 98.04% and an F1-score of 0.98 in the detection of human actions. Moreover, Bidirectional LSTM (BiLSTM) enhances the functionality of conventional LSTM by analysing data in both the forward and backward directions, thus capturing a greater amount of contextual information. In 2022, Challa et al. [40] proposed a hybrid model that combines CNN with BiLSTM. This model achieved impressive accuracy rates on many datasets, such as WISDM (96.05%), UCI-HAR (96.37%), and PAMAP (94.29%). The inclusion of the BiLSTM component enabled the model to comprehensively grasp the complete context of activity sequences, hence improving the performance of categorization.

Additionally, researchers have been exploring the potential of hybrid DL models to further improve accuracy and efficiency. For example, the CNN-LSTM architecture proposed by Mutegeki and Han [42] effectively reduced model complexity and achieved an accuracy of 99.00% on an internal dataset and 92.00% on the UCI-HAR dataset. Similarly, the study by Semwal et al. [43] focused on recognizing walking activities using IMU sensors, introducing four hybrid DL models that achieved a classification accuracy of 99.34%. GRUs, which have a reduced design compared to LSTM, are used to decrease computational complexity without sacrificing performance. GRUs possess a lower number of parameters compared to LSTM, rendering them less susceptible to overfitting and enabling faster training. For example, Zhu et al. [31] employed CNN to extract features and GRU for sequence modelling. This approach resulted in a classification accuracy of 96.11%. The hybrid technique effectively resolved challenges such as varying motion modes and limited data availability. Moreover, a novel deep neural network architecture that integrated LSTM with convolutional layers was introduced in [39]. This fusion of LSTM and convolutional layers yielded a model characterised by reduced parameter count and remarkable accuracy. Specifically, the suggested model achieved an impressive accuracy rate of 95.78% when evaluated on the UCI-HAR dataset. Finally, to tackle the issue of implementing DL algorithms on

TABLE I. Summary of identified studies employing DL methods for domains of physical health assessment through the use of wearable data.

S/D	Study	Year	Sensor/ Device	DL method	Dataset/Stimuli	Performance
ADL	Dua et al. [30]	2021	IMU	CNN-GRU	UCI-HAR, WISDM, PAMAP	Acc. = .97, f1 = .97
	Zhu et al. [31]	2019	IMU	CNN	In-house (ADL activities)	Acc. = .96
	Mohamed et al. [32]	2022	IMU, HR	CNN	PAMAP, Extra-sensory	Acc. = .68
	Teng et al. [33]	2020	IMU	CNN	UCI-HAR, OPPOETUNITY, UniMib-SHAR, PAMAP, WISDM	Acc. = .99, f1 = .99
	Xing et al. [34]	2019	EEG	CNN	In-house (driving)	Acc. = .82
	Kuncan et al. [35]	2022	IMU	LSTM	Daily and Sport Activities Dataset	Acc. = .98
	Sarkar et al. [36]	2023	IMU	SA-CNN	UCI-HAR, WISDM, MHEALTH, PAMAP2, HHAR	Acc. = .98
	Dirgová et al. [37]	2022	IMU	Transformer	KU-HAR	Acc. = .99
	Mekrusavanich et al. [38]	2022	IMU, GPS, light, sound sensor	RLSTM	REALWORLD16	Acc. = .98, f1 = .98
	Xia et al. [39]	2020	IMU	CNN-LSTM	UCI-HAR, WISDM, OPPORTUNITY	Acc. = .96
	Challa et al. [40]	2022	IMU	BiLSTM-CNN	WISDM, UCI-HAR, PAMAP	Acc. = .96, f1 = .96
	Agarwal et al. [41]	2020	IMU	RNN-LSTM	In-house (ADL activities)	Acc. = .96
	Mutegeki et al. [42]	2020	IMU	CNN-LSTM	iSPL, UCI-HAR	Acc. = .99
	Semwal et al. [43]	2021	IMU	CNN-LSTM	MHEALTH	Acc. = .99
MA	Odhiambo et al. [44]	2023	IMU	DNN	In-house (medication taking)	Pr. = .96, Rec. = .94
	Lee et al. [45]	2021	IMU, Wearable camera	CNN	In-house (medication taking)	Acc. = .93, Pr. = .91, Rec. = .95
	Ntalianis et al. [46]	2019	Inhaler	CNN	In-house (inhaling procedure)	Acc. = .95
	Pettas et al. [47]	2019	Inhaler	LSTM	In-house (inhaling procedure)	Acc. = .94
	Thyde et al. [48]	2021	Glucose monitor	CNN	In-house	Acc. = .77
	Wu et al. [49]	2021	EEG	CSAE	In-house (flight simulation)	Acc. = .83
	Bai et al. [50]	2020	EEG, Actigraphy	LSTM-CSA	In-house (fatigue level recording)	MAE = 1.39
EF	Guan et al. [51]	2021	ECG, IMU	BiLSTM	In-house (running)	Acc. = .81
	Wang et al. [52]	2023	EEG	CNN	In-house (construction operation)	Acc. = .89
	Budak et al. [53]	2019	PPG, EEG	CNN	MIT/BIH	Acc. = .94
	Wang et al. [54]	2023	IMU	TCN-MHA	FD I&II	MAE = .11
	Utomo et al. [55]	2019	HRV, Eye-tracker	RNN-LSTM	In-house (driving simulation)	Acc. = .88
	Balam et al. [56]	2021	EEG	CNN	SLEEP-EDF	Acc. = .95
	Sharma et al. [57]	2022	IMU	CNN	Clemson all-day	Acc. = .89
	Lee et al. [58]	2019	ECG, PPG	CNN	In-house (driving simulation)	Acc. = .64, Pr. = .71, Rec. = .78, f1 = .71

S/D	Study	Year	Sensor/ Device	DL method	Dataset/Stimuli	Performance
Mobility	Kulurkar et al. [59]	2023	IMU	LSTM	MobiAct	Acc. = .96
	Yang et al. [60]	2024	IMU	Temp. CNN	In-house	ICC = .92
	Mo et al. [61]	2023	IMU	Tr. BiLSTM	Defog dataset	MAP = .43
Pain	Gkikas et al. [62]	2023	ECG	MT-NN	BioVid Heat Pain	Acc. = .69
	Rojas et al. [63]	2021	fNIRS	BiLSTM	In-house (pain invoking)	Acc. = .92, Sens. = .85, Spec. = .90
	Pouromran et al. [64]	2022	EDA	BiLSTM	In-house (cold pressor test)	f1 = .81, AUC = .93
	Hu et al. [65]	2018	MoCap, EMG	LSTM	In-house (balance test)	Pr. = .97, Rec. = .97
	Wang et al. [66]	2021	MoCap, EMG	LSTM	EmoPain	Acc. = .83, Pr. = .81, Rec. = .83, f1 = .82
	Wang et al. [67]	2020	EEG	CNN-AE	In-house (pain invoking)	Acc. = .75
	Yu et al. [68]	2020	EEG	DFB-CNN	In-house (pain invoking)	Acc.= .97
	Lim et al. [69]	2019	PPG	DBN	In-house (surgical operation)	Acc. = .87
	Arenas et al. [70]	2023	EDA	CNN-LSTM	ChonLab Thermal Grill	Acc. = .93, Sens. = .82, Spec. = .95, f1 = .78
Sleep	Kilic et al. [71]	2023	PPG	CNN	Nethealth	MAE = .04
	Arora et al. [72]	2020	PPG, Actigraphy	CNN	Inhouse, MESA	Acc. = .91, Pr. = .90, Rec. = .92, f1 = .91
	Yildirim et al. [73]	2019	EEG	CNN	SLEEP-EDF, SLEEP-EDFX	Acc. = .98
	Mousavi et al. [74]	2019	EEG	CNN	SLEEP-EDF	Acc. = .84, f1 = 0.80
	Supratak et al. [75]	2017	EEG	CNN-LSTM	SLEEP-EDF, MASS	Acc. = .86
	Sathyanarayana et al. [76]	2016	Actigraphy	CNN	In-house (sleeping procedure)	Sens. = .97, Spec. = .86, AUC = .94
	Phan et al. [77]	2020	PPG	LSTM	In-house (sleeping procedure)	Acc. = .61, Sens. = .72, Spec. = .49, AUC = .61
	Matsumori et al. [78]	2022	EEG	CNN-LSTM	In-house (sleeping procedure)	Acc. = .79, f1 = .74

ADL: Activities of Daily Living, MA: Medication Adherence, EF: Energy and Fatigue, IMU: Inertial Measurement Unit, HR: Heart Rate, EEG: Electroencephalogram, GPS: Global Positioning System, ECG: Electrocardiogram, PPG: Photoplethysmogram, MoCap: Motion Capture device, EMG: Electromyography, fNIRS: Functional Near-Infrared Spectroscopy, EDA: Electrodermal Activity, CNN-GRU: CNN with Gated Recurrent Unit, CSAE: Convolutional and Self-Attention Encoder, LSTM-CSA: Long Short-Term Memory with Consistency Self-Attention, CNN-AE: Convolutional Neural Network with Autoencoder, DFB-CNN: Depthwise Separable Convolutional Neural Network, DBN: Deep Belief Network, TCN-MHA: Temporal Convolutional Network with Multi-head Attention. ICC: Intra-class Correlation Coefficient

edge devices, another study proposed a Lightweight DL Model (RNN-LSTM) for HAR trained on WISDM dataset, achieving high performance on participants' daily activities data [41], reaching an accuracy of 95.78%.

However, recent progress in the processing of time-series data has led to a transition from traditional DL architectures to more advanced variations. A prominent pattern is the utilisation of CNN-Attention models, which augment the capacity to concentrate on significant characteristics within the chronological sequence of sensor data. In a relevant study in [36],authors have proposed a hybrid architecture for HAR that utilises wearable sensor data. The process commenced by transforming the time series data from the sensors into multichannel pictures through the utilisation of Continuous Wavelet Transform. Subsequently, a CNN with Spatial Attention (SA-CNN) was utilised to extract features of a high-dimensional nature. A unique attribute selection technique has been created to find crucial criteria for identifying human behaviours. Initially, three filter-based strategies, namely Mutual Information (MI), Relief-F, and minimum redundancy maximum relevance (mRMR), were employed to evaluate the appropriateness of features. An adapted version of the Genetic Algorithm (GA) was also utilised to identify the most optimal set of features by removing features with lower rankings. Finally, the K-Nearest Neighbours (KNN) classifier was employed to classify human activities. The proposed model has been tested extensively on five publicly accessible HAR datasets (UCI-HAR, WISDM, MHEALTH, PAMAP2, and HHAR), achieving an average accuracy of 97.72%.

Another significant development is the application of transformer models in processing wearable data. Such a study in [37] involved adapting a transformer model for the purpose of analysing motion signals in a time-series format. The self-attention mechanism present in the transformer allowed for the expression of individual dependencies between signal values in a time series. The performance of the proposed adapted transformer method was evaluated using the KU-HAR dataset of smartphone motion sensor data, which encompasses a diverse range of activities. The method achieved an average identification accuracy of 99.20%, surpassing the 89.67% accuracy achieved by a conventional machine learning method on the same dataset.

B. Medication Adherence (MA)

Medication adherence refers to the degree to which patients follow the prescribed medication regimen, including the correct dosage, timing, and duration of treatment as recommended by their healthcare providers [80]. Non-adherence to healthcare management is a crucial component due to its potential to result in treatment ineffectiveness, escalated healthcare expenditures, and an elevated likelihood of adverse events. The utilisation of emerging technologies and advanced DL techniques has been progressively significant in the assessment and evaluation of medication adherence, owing to their capacity to offer enhanced precision, immediate feedback, and tailored observations [81].

Several notable surveys have made essential contributions to the investigation of utilising wearable data to improve the evaluation of medication adherence. A recent study [44] investigated the detection of involuntary movements that frequently occur with the administration of medication using a Deep Neural Network (DNN). By utilising smartwatches, the researchers collected accelerometer data at a frequency of 25 Hz. They were able to achieve a precision of 96.50% in identifying positive results and 94.50% in identifying negative outcomes. This study emphasises the capability of DNN in analysing time-series data obtained from wearable sensors to enhance the monitoring of adherence.

CNN have been successfully utilised in the context of medication adherence. Lee et al. [45] suggested combining a camera image sensor with a wearable device to record the process of taking medication. Through the utilisation of CNN for picture processing, their approach attained a recognition accuracy of 92.70% in identifying medication behaviour. This showcases the capability of CNN to effectively process intricate visual data with conventional sensor data. Additionally, Ntalianis et al. [46] utilised a deep sparse CNN to evaluate real-time medication adherence in chronic inflammatory pulmonary illnesses by analysing inhaler usage data. This method effectively decreased computing complexity while still achieving a high classification accuracy of 95.00%. In a more recent study, Thyde et al. [48] conducted a study in a specific healthcare environment where they aimed to create an algorithm that can accurately identify whether patients with type 2 diabetes (T2D) are following their prescribed treatment using DL techniques. Through the simulation of continuous glucose monitoring (CGM) signals and the comparison of several categorization algorithms, researchers determined that CNN were the most efficient method, with an average accuracy of 77.50%.

Researchers in [47], employed LSTM networks in conjunction with spectrogram characteristics to detect audio events originating from pressurised metered dose inhalers. The efficacy of LSTM in handling temporal dependencies in audio data is highlighted by the superior performance achieved by this method, surpassing that of typical machine learning techniques, with accuracies ranging from 92.00% to 94.00%.

C. Energy and Fatigue (EF)

Energy and fatigue (EF) are fundamental elements of an individual's physiological condition that exert a direct influence on their general welfare and performance [82]. Energy can be defined as the inherent ability, both physically and mentally, to effectively engage in various activities. Conversely, fatigue is a contrasting state characterised by feelings of weariness, exhaustion, or the depletion of energy reserves [82]. Accurate measurement of EF is of utmost importance in comprehending an individual's health status, maximising productivity, and improving overall QoL.

Energy levels play a crucial role in an individual's overall well-being and daily functioning. Sharma et al. [57] presented a novel technique for identifying instances of eating by tracking wrist movement throughout everyday tasks. The authors utilised a CNN to examine significantly extended

time intervals, varying from 0.5 to 15 minutes, enabling the incorporation of further hand movements related to eating. This approach was verified using the publicly available Clemson all-day dataset. The proposed technique, while using a 6-minute window, produced a detection rate of 89.00% for eating events.

With the emerging need for advanced DL models to address complex health-related challenges, there is a growing interest in studying the correlation between eating speed and several intake-related issues, including obesity and diabetes. To detect bites from free-living IMU data, a temporal CNN paired with a multi-head attention module (TCN-MHA) was utilised [54]. The anticipated sequences of bites were subsequently grouped into eating episodes. In order to verify the effectiveness of the suggested method for measuring eating speed, a 7-fold cross validation was conducted using a self-collected dataset (FD I&II), which contains detailed annotations for a whole day of eating activities. The experimental findings demonstrated that the proposed strategy attained a MAE of 0.11.

Research on the identification of mental stress and fatigue is of utmost importance due to its significant impact on performance and safety. Several DL methods have been used to improve the accuracy and resilience of these detection systems. Wu et al. [49] proposed a Contractive Sparse Auto-encoder model that utilises power spectrum analysis of electroencephalogram (EEG) signals to detect pilot tiredness states. The model achieved an accuracy of 83.00% in recognising these states. The proficiency of this model in compressing and reconstructing input data while maintaining crucial characteristics showcases the power of auto-encoders in handling high-dimensional EEG data.

Self-attention processes have played a crucial role in the identification of weariness. Bai et al. [50] devised an automated approach that employs self-attention models and feature selection to evaluate fatigue based on Electrocardiograph (ECG) and Actigraphy data gathered in unrestricted settings. The LSTM-CSA approach, which combines LSTM's temporal modelling capabilities with self-attention's capacity to focus on essential characteristics over long sequences, achieved a Mean Absolute Error (MAE) of 1.39, showcasing its effectiveness. Guan et al. [51] in the field of sports science created a wearable device to identify exercise tiredness. This was achieved by collecting ECG signals, acceleration, and angular velocity. A BiLSTM was utilised to categorise three stages of sports fatigue, resulting in a user-independent recognition accuracy of 80.55%. The bidirectional feature of BiLSTM allows the model to gather information from both preceding and subsequent contexts in the data, which is essential for accurately detecting stages of fatigue.

Wang et al. [52] used a CNN architecture to analyse EEG data in order to address mental weariness in construction workers. They achieved an accuracy rate of 88.85%. The capability of CNN to extract hierarchical features from raw EEG signals makes them highly suitable for this task, as it requires the identification of delicate and intricate patterns for precise fatigue detection.

In the realm of road safety and driver drowsiness detection, Budak et al. [53] employed a pre-trained CNN to extract deep features and paired it with a customisable Q-factor wavelet transform. This approach resulted in an average accuracy rate of 94.31%. This approach showcases the effectiveness of pre-trained CNN in utilising transfer learning to extract significant features from intricate datasets. Additionally, the wavelet transform accurately captures the temporary attributes of sleepiness signals. In another investigation conducted by Utomo et al. [55], an RNN with LSTM units was utilised to detect driver fatigue. The input variables for this detection included Heart Rate Variability (HRV) and the Percentage of Eyelid Closure (PERCLOS). The model successfully achieved an accuracy of 88.00% by utilising the LSTM's capacity to capture temporal relationships and make predictions about future states based on past data. This was specifically applied to forecasting the probability of driver fatigue over time. Lee et al. [58] also examined HRV signals obtained from wearable ECG and photoplethysmogram (PPG) sensors to detect driver tiredness. The researchers employed CNN with Rectified Linear Unit with Randomised Projections (ReLU-RP) activation functions to categorise states of drowsiness and wakefulness. They observed a notable enhancement in accuracy, ranging from around 6% to 17% for ECG data and 4% to 14% for PPG data, when compared to earlier investigations.

D. Mobility

Within the realm of global and public health, the concept of mobility has been delineated by the International Classification of Function, Disability and Health (ICF) established by the WHO [83]. This classification encompasses various elements including altering and sustaining bodily positions, transporting, manipulating and relocating objects, ambulation and locomotion, as well as utilising transportation for movement purposes. The definition utilising highlights the evident fact that mobility is an essential capacity, albeit not the sole determinant, that is indispensable for various activities encompassing basic activities of daily living, more intricate activities necessary for self-care and managing living arrangements, occupational pursuits, and engagement in recreational and leisurely pursuits, including sports. The downstream activities that rely on a certain level of mobility are significant contributors to an individual's QoL.

Given the multifaceted definition of mobility, its quantification can indeed be a challenging task. By leveraging the integration of GPS technology in wearable devices, it becomes feasible to compute life-space mobility [84]. This metric pertains to the extent of a person's movement away from their residence and has been demonstrated to correlate with factors such as social support, driving ability, and gait speed [85]. Ranked second on the list, this factor provides valuable insights into the individual's QoL by assessing the frequency of strenuous physical activities through the measurement of heart rate and physical accelerations. Wearable accelerometers additionally offer data regarding the velocity of motion, including step cadence and the duration of activity intervals. Many smartphone and smartwatch apps are capable of monitoring and recording higher levels of physical activity, such as those attained through stair climbing [86]. The Timed-Up-and-Go (TUG) test, as demonstrated in another clinical study, exhibits

significance in assessing QoL [87]. This assessment is reflective of an individual's mobility since it entails the execution of specific movements, including rising from a seated position, ambulating a distance of 3 metres, performing a turn, and afterwards returning to a seated posture.

The potential for experiencing a fall can lead to a significant decrease in the mobility, autonomy, and overall well-being of an older individual [22]. Among the several studies focused on fall risk assessment, there is a notable IoT-based system that utilises low-power wireless sensing networks, big data, cloud computing, smart devices, and DL to identify falls of elderly individuals in indoor settings [59]. A wearable sixLowPAN gadget incorporated a three-dimensional axis accelerometer to collect real-time data on the movements of elderly individuals. The sensor signals were processed and analysed using a LSTM model on an advanced Internet of Things (IoT) gateway to achieve optimal accuracy in detecting falls. The authors investigated the optimal Nyquist rate, sensor placement, and information exchange over several channels using the training set derived from the public dataset ”MobiAct”. Their edge computing solution achieved a 95.87% accuracy rate in detecting falls by utilising real-time data stream analytics.

Freezing of gait (FOG), a episodic and extremely debilitating sign of Parkinson's Disease (PD), is often evaluated by time-consuming eye examination. Existing machine learning-based FOG prediction models provide excellent sensitivity and specificity when using time-series data [22], [60]. However, these models do not provide detailed information about the precise types of FOG events. In order to tackle this problem, a novel study conducted in [61], utilised a transformer encoder architecture combined with Bidirectional LSTM layers to create multiple deep learning models. These models were designed to capture long-range dependencies in sequential data and used different feature sets to predict three distinct types of FOG events using acceleration data. The top-performing model attained a Mean Absolute Precision (MAP) of 0.43.

Nevertheless, if we were to use mobility to quantify QoL, we should consider integrating these sensor-based metrics with other QoL indicators and potentially using complementary data sources, such as surveys, interviews, or self-reports, to gather information on the broader aspects of mobility that are difficult to capture solely through wearable sensors. Notably, often the terms mobility and activity are used interchangeably, which can lead to confusion, but they refer to different aspects of a person's movement and behavior [88]. While activity recognition is a component of mobility assessment, it's just one part of the broader picture. As aforementioned, mobility assessment considers not only the specific activities but also the context in which they occur, the mobility limitations an individual may face, and how mobility relates to their overall QoL. This multidimensional approach can provide a more comprehensive and accurate assessment of how mobility influences an individual's overall QoL. However, in this review we focus on wearable technologies and therefore, we assume that the subset of mobility that can be directly quantified through DL utilizing data from wearable sensors is included in the assessment of ADL as well as EF, presented in Sections II-A and II-C respectively.

E. Pain

The perception of pain is a multifaceted and subjective sensory phenomenon that exhibits variations in both intensity and quality, hence posing difficulties in its objective quantification [62]. The measurement of human health is of paramount importance, as it functions as a reliable marker for latent medical disorders, informs the process of clinical decision-making, and evaluates the effectiveness of treatments. In response to the requirement for enhanced precision and impartiality in pain evaluation, recent advancements in wearable technologies, such as electrodermal activity (EDA) sensors, in conjunction with DL techniques, have emerged as potentially valuable instruments for the quantification and measurement of pain, an asset of QoL.

The subject of autonomous pain quantification utilising DL techniques is expanding rapidly and has important implications for patient care and monitoring. Gkikas et al. [89] conducted a study where they investigated the application of multi-task learning (MTL) neural networks to quantify pain using ECG inputs. They utilised the Pan-Tompkins algorithm to identify QRS complexes and get Inter-Beat Intervals (IBIs). Subsequently, they constructed a MTL model that integrated pain evaluation with gender and age prediction tasks. The utilisation of a multi-task strategy resulted in more advantageous results when compared to standard single-task methods. This highlights the benefit of utilising shared representations in MTL to improve the performance of models across related tasks.

Rojas et al. [63] tackled the challenge of evaluating pain in patients who cannot communicate verbally by employing functional near-infrared spectroscopy (fNIRS) and a BiLSTM model. Their methodology demonstrated a remarkable accuracy rate of 90.60%, underscoring the promise of BiLSTM in effectively analysing sequential data from fNIRS to precisely evaluate pain conditions. In a similar vein, Pouromran et al. [64] examined the categorization of pain intensity by employing a tailored BiLSTM model in conjunction with an ensemble technique. Their approach surpassed traditional methods, attaining an average f1-score of 0.81 and an Area Under the Receiver Operating Characteristic curve (AUROC) of 0.93 for four pain states: no pain, moderate pain, medium pain, and extreme pain. The ensemble technique, which combines many models to enhance resilience and precision, shown efficacy in addressing the intrinsic heterogeneity in pain perception and expression.

In their study, Hu et al. [65] concentrated on the detection of chronic low back pain (LBP) by analysing balance and body sway. They utilised a motion tracking system based on an electromagnetic field. Their LSTM model exhibited precision and recall rates of 97.20%, highlighting the efficacy of LSTM in modelling time-series data to differentiate between LBP and non-LBP populations. Lastly, Wang et al. explored the independent recognition of protective behavior (PBD) across various activities [66]. Their study employed stacked LSTM techniques in the analysis of data acquired from wearable

motion capture devices and EMG measures, demonstrating a notable level of agreement with expert evaluations. The optimal performance achieved a mean f1-score of 0.82 by the utilisation of leave-one-subject-out cross validation. When protective behaviour was demonstrated based on the type of activity, the average f1-scores for performance were as follows: 0.77 for bend-down, 0.81 for one-leg-stand, 0.72 for sit-to-stand, 0.83 for stand-to-sit, and 0.67 for reach-forward.

The research conducted by Wang et al. [67] investigated the application of EEG signals for the purpose of objectively evaluating pain. The researchers utilised a CNN-based autoencoder model, which outperformed traditional methods by achieving an accuracy of 74.60%. This highlights the effectiveness of autoencoder models in extracting features and reducing dimensionality, which is crucial for dealing with the high-dimensional EEG data. Yu et al. [68] extended the use of CNN by introducing variable frequency band-based CNN (DFB-based ConvNets) to differentiate between different cold pain states. Their model demonstrated an exceptional classification accuracy of 97.37%, highlighting the significance of frequency band analysis in improving the performance of CNN for pain detection.

Lim et al. (2019) conducted a study that specifically examined the evaluation of pain during surgical procedures. They emphasised the importance of accurately assessing pain in real-time clinical environments. They employed a deep belief network (DBN) in conjunction with PPG signals to accurately classify pain states. Their model outperformed conventional approaches, obtaining an accuracy of 86.79% for two-class pain condition classification and 65.67% for four-class pain status classification. Arenas et al. [70] emphasised the subjective nature of pain appraisal and the importance of continuous, objective observation. Their study examined various deep learning models, such as 1D-CNN, LSTM, and hybrid CNN-LSTM architectures, to analyse and detect ongoing discomfort by utilising EDA data. The hybrid model, which combines the spatial feature extraction capabilities of CNN and the ability of LSTM to capture temporal dependencies, achieved a f1-score of 0.78 for signal segments lasting 15 seconds. This method emphasises the benefits of hybrid models in managing multimodal time-series data for the purpose of continuous pain monitoring.

F. Sleep

Sleep is an essential physiological condition characterised by a momentary cessation of awareness, diminished sensory activity, and modified brain functioning [90]. It normally follows a cyclical pattern consisting of many sleep stages, including REM (rapid eye movement) and non-REM stages. Sleep is of utmost importance in the preservation of both physical and mental well-being, as it serves as a vital component for restorative processes, memory consolidation, and overall holistic health [80]. Thus, incorporating emerging wearable technologies and advanced DL methodologies in the quantification and assessment of sleep holds significant significance. This is because it not only facilitates individuals in acquiring enhanced understanding of their sleep patterns, but also aids healthcare practitioners in the diagnosis of sleep disorders, optimisation of treatment strategies, and promotion of healthier sleep habits.

The assessment of sleep quality can be effectively conducted by utilising data obtained from wearable devices, as exemplified by the NetHealth dataset [71]. The dataset employed in this study comprises data obtained from a sample of 698 college students, utilising both wearables and surveys as data collection methods. Sleep data analysis utilised different architectures of CNN in order to evaluate sleep quality with the best achieving a MAE of approximately .04. CNN were also tested for the objective to forecast sleep quality by utilising data collected from wearable devices, as outlined in the research conducted by [72]. Three sleep indicators were obtained from wearable data: Daily Sleep Quality, Weekly Sleep Quality, and Sleep Consistency. CNN had superior performance compared to multilayer perceptrons (MLP), attaining an accuracy rate of 97.30%.

The manual rating of polysomnogram (PSG) signals is a labor-intensive and error-prone process, as discussed by Yildirim et al. [73]. In order to tackle these difficulties, they employed an innovative 1D-CNN model to categorise sleep stages by utilising unprocessed PSG data. The results of this approach revealed remarkable levels of accuracy, ranging from 91.00% to 98.06% across different datasets. In the same year, Mousavi et al. [74] proposed the SleepEEGNet model, which utilises single-channel EEG inputs to automatically categorise sleep stages. SleepEEGNet obtained an accuracy of 84.26% by using a mix of CNN and sequence-to-sequence models. This study emphasises the efficacy of combining convolutional layers with sequence models to capture spatial and temporal characteristics in EEG signals, hence improving the model's capacity to distinguish between different sleep stages. Similarly, the DeepSleepNet model, as introduced by Supratak et al. [75], sought to automate the classification of sleep stages by utilising unprocessed EEG data from a single channel. DeepSleepNet, which utilised a combination of CNN and BiLSTM networks, outperformed earlier models by obtaining an accuracy of 86.20%. This strategy leverages the CNN's capacity to extract significant features and the BiLSTM's ability to capture long-term dependencies in the EEG data, resulting in a strong solution for sleep stage classification.

Actigraphy sensors are of significant importance in the prediction of sleep efficiency [76]. DL models, such as MLP, CNN, and LSTM were tested in this study, with the CNN demonstrating the highest level of accuracy, reaching an impressive rate of 97.30%. This study aimed to identify the primary factors that have a significant impact on the quality of sleep. Additionally, Phan et al. emphasised the utilisation of DL models for the purpose of forecasting sleep quality [77]. The data obtained from the utilisation of smartwatch devices by students were subjected to analysis. Among different trained machine learning methods such as CNN and GRU, LSTM demonstrated efficacy in predicting sleep quality by leveraging data on physical activities during waking with 61.00% accuracy.

A further investigation is centred around the application

of EEG data for the purpose of sleep-stage classification, employing the Haru EEG sensor as the primary instrument [78]. The utilisation of a lightweight and economically viable sensor, in conjunction with a hybrid two channel CNN-LSTM model, resulted in 78.60% accuracy that is comparable to that of clinical PSG equipment.

G. Work Capacity

According to the American College of Sports Medicine (ACSM), work capacity is the greatest amount of physical work or exercise that an individual can accomplish [91]. It is commonly evaluated in terms of power output or endurance and is affected by physiological factors including cardiorespiratory fitness, muscular strength, and endurance. The aforementioned criterion plays a crucial role in evaluating an individual's physical fitness and general health, while also providing guidance for exercise prescription and tracking the progress of training.

Historically, several study investigations pertaining to physical activity and work capability have heavily depended on self-report tools. Nevertheless, subjective assessments of activity are prone to significant unreliability due to factors such as memory, social desirability, and various biases. Consequently, these subjective measures may yield values that are either higher or lower than those obtained by objective measurements [92]. The Work-ability Support Scale (WSS) is a recently developed instrument that aims to evaluate an individual's vocational capacity and support requirements after the occurrence of an acquired disability [93]. This tool is intended for utilisation by individuals who are currently employed, as well as those who are contemplating reentering the workforce, serving as a planning aid. The instrument encompasses a total of 16 items that are distributed throughout three distinct domains of work performance, namely physical/environment, thinking and speaking, and social/behavioral. The scoring system spans from 1, indicating constant help, to 7, indicating independence. In addition, there exist seven other elements pertaining to contextual circumstances beyond the confines of the workplace that possess the potential to impact work performance. The scoring accuracy and rater reliability of the system have received empirical validation.

Furthermore, a Functional ability Evaluation (FCE) is an assessment that examines an individual's functional ability in relation to the physical and cognitive demands of a specific employment [94]. The FCE encompasses many components such as lifting, carrying, pushing/pulling, balance, fine motor skills, and cardiovascular endurance. The determination of capacity for these tasks is based on the Dictionary of Occupational Titles (DOT) published by the US Department of Labour. Moreover, the Work Ability Index (WAI) is a tool employed in clinical occupational health and research settings to evaluate job capacity as part of health assessments and workplace investigations [95]. The determination of the index is based on a set of questions that examine factors such as work demands, the health status of the worker, and available resources. The employee diligently fills out the questionnaire prior to the interview with an occupational health specialist, who assesses the answers based on the provided guidelines. Additionally, the Work Capacity Test (WCT), sometimes referred to as the pack test, is a physical examination originally administered by the U.S. Forest Service to evaluate the physical capabilities of wildland firefighters [96]. The pack test is deliberately designed to induce stress in order to assess the firefighter's muscular strength and aerobic endurance capabilities. The pack test possess great validity in assessing an individual's capacity to engage in strenuous physical work over an extended duration, such as 45 minutes. In addition to the U.S. Forest Service, the majority of federal, state, and private agencies employ the pack test as their preferred physical fitness assessment.

The assessment of work capacity necessitates the evaluation of the alignment between the physical, mental, social, environmental, and organisational requirements of an individual's job and their ability to fulfil these requirements. The assessment of workability necessitates the examination of various elements, encompassing the individual's physical capacity to carry out tasks, their ability to handle the cognitive and communicative requirements of the job, as well as their aptitude to operate effectively within the social and environmental framework of the workplace. Wearable activity trackers include the capability to quantify numerous elements of work capacity, rendering them particularly advantageous for persons engaged in physically demanding occupations. Nevertheless, similarly to the case of mobility in Section II-D, we assume that the subset of work capacity that can be quantified through DL utilizing directly data from wearable sensors is strongly correlated with the assessment of ADL, presented in Section II-A.

III. Psychological health

The QoL of an individual is significantly influenced by their physiological health, which includes various dimensions such as feelings self-esteem, memory, spirituality, and body image [97]. The importance of physiological health within the larger framework of QoL becomes apparent when we take into account its direct influence on many domains. This section will explore the emerging area of DL in the context of assessing physiological health by utilising data collected from wearable devices. Through an examination of current research and methodology, our objective is to shed light on the crucial significance of DL in enhancing our capacity to measure and evaluate physiological well-being. This, in turn, will contribute to a more thorough comprehension of QoL within various cultural and contextual settings. The summary of the identified studies is presented in Table II.

A. Feelings

Feelings, which include a range of both negative and positive affective states, constitute a basic subset within the domain of physiological well-being [118]. The complex range of emotional states, which encompass both positive emotions such as joy and contentment, as well as negative emotions like tension and anxiety, are fundamental elements that contribute to an individual's holistic well-being and overall QoL [119]. In recent times, there have been notable breakthroughs in the field of wearable technology, which have expanded the possibilities for the recognition of feelings and emotions.

TABLE II. Summary of identified studies employing DL methods for domains of psychological health assessment through the use of wearable data.

S/D	Study	Year	Sensor/ Device	DL method	Dataset/Stimuli	Performance
Feelings	Skaramagkas et al. [98]	2023	Eye-tracker	DNN	eSEE-d	Acc. = .92, f1 = .91, AUC = .91
	Wang et al. [99]	2020	EDA, SKT, PPG	DNN	In-house (emotion evoking videos)	Acc. = .74, Pr. = .74, Rec. = .77, AUC = .77
	Dissanayake et al. [100]	2022	IMU, EDA, ECG, PPG, BVP, SKT	CNN	AffectiveROAD, CASE, CLAS, K-EmoCon	Acc. = .81
	Zeng et al. [101]	2020	EEG, ECG	LSTM	In-house (emotion evoking videos)	Acc. = .95
	Nakisa et al. [102]	2018	EEG, BVP	LSTM	In-house (emotion evoking videos)	Acc. = .78
	Tu et al. [103]	2023	Mobile phone	MKFM	MELD, IEMOCAP, EmoryNLP	f1 = .68
	Tizzano et al. [104]	2020	IMU, HR	LSTM	In-house (emotion evoking videos) [105] (emotion evoking videos)	Acc. = .71
	Lian et al. [106]	2021	Wearable camera	CNN-LSTM	In-house (emotion evoking videos)	Acc. = .99, Pr. = .99, Rec. = .99, f1 = .99
	Kanjo et al. [107]	2019	HR, GSR, SKT, IMU, UV, GPS	CNN-LSTM	envbodysens	Acc. = .95, f1 = .95
	Chen et al. [108]	2020	EEG	RNN-CNN	DEAP	Acc. = .93
Self-esteem	Buettner et al. [109]	2021	EEG	CNN	No information provided	Acc. = .79
Thinking	Gupta et al. [110]	2021	EEG	CNN	In-house (N-back task)	Acc. = .81
	Gasparini et al. [111]	2021	PPG	CNN	In-house (mentally demanding tasks)	Acc. = .79
	Almogbel et al. [112]	2021	EEG	CNN	In-house (driving simulation)	Acc. = .97, Pr. = .90, Rec. = .87, f1 = .88
	You et al. [113]	2021	EEG	DNN	In-house (mentally demanding tasks)	Acc. = .81, Sens. = .67, Spec. = .95
	Kaczorowska et al. [114]	2021	Eye-tracker	DNN	In-house (eye activity)	Acc. = .97, Pr. = .98, Rec. = .97, AUC = .99, f1 = .97
	Kaushik et al. [115]	2022	EEG	LSTM	In-house (mentally demanding tasks)	Acc. = .96
	Yang et al. [116]	2019	EEG	SDAE	Yin et al. [117] (mentally demanding tasks)	Acc. = .92

EDA: Electrodermal Activity, SKT: Skin Temperature, PPG: Photoplethysmogram, IMU: Inertial Measurement Unit, ECG: Electrocardiogram, BVP: Blood Volume Pulse, EEG: Electroencephalogram, HR: Heart Rate, GSR: Galvanic Skin Response, UV: Ultraviolet (Sensor), GPS: Global Positioning System, SDAE: Stacked Denoising Autoencoder, MKFM: Multiple Knowledge Fusion Model.

Wearable sensors have emerged as indispensable instruments for the real-time evaluation of emotional states, as they possess the capability to capture physiological signals including heart rate variability, skin conductance, facial expressions, and even eye movements [120]–[122]. This section aims to examine the dynamic development of DL methods developed specifically for the purpose of recognising feelings. It will provide insight into the potential of these methods and sensors to significantly transform our comprehension of emotional well-being and its substantial influence on QoL.

In recent years, there has been an increasing scholarly focus on the assessment of affective states. This surge in interest can be attributed to the progress made in data analysis through the utilisation of artificial intelligence techniques, as well as the ubiquitous availability of high-quality video material. Nevertheless, the scarcity of comprehensive benchmark datasets has compelled researchers to develop novel methodologies. An example of a resource that can be utilised is the eSEE-d database, which employs data from a wearable eye-tracking device to approximate emotional states of individuals who are exposed to emotional videos and afterwards complete self-assessment surveys [98]. The Deep Multilayer Perceptron (DMLP) networks demonstrated notable accuracy, achieving a positive valence discernment rate of 92.00% and a low arousal identification rate of 81.00%. The dataset used in this study has been made publicly accessible. Moreover, the proliferation of smart wearables has enabled the acquisition of physiological and behavioral data for recognizing human behaviors and emotions. To achieve precise emotion recognition, authors in [99] proposed an adaptive system using a sensor-enriched smartwatch. It employed multi-mode sensory data (including blood volume pulse (BVP), EDA, and skin temperature (ST)) for emotion detection (arousal and valence), resulting in a 74.30% accuracy rate among participants by employing a DNN.

Wearable smart devices have revolutionized the acquisition of physiological data for emotion recognition, yet maintaining data integrity and the scarcity of labeled data remain significant challenges. Dissanayake et al. [100] addressed these issues by presenting a self-supervised CNN-based approach inspired by contrastive learning. By leveraging physiological signals from four different datasets, their methodology achieved an impressive 81.00% accuracy in emotion recognition and demonstrated remarkable resilience to data degradation. This study emphasizes the potential of self-supervised learning techniques in enhancing model performance and robustness in scenarios with limited labeled data.

LLMs, such as ChatGPT, have exhibited proficiency in emotion recognition, especially in the analysis of textual material from conversational and narrative contexts. Tu et al. [103] introduced a Multiple Knowledge Fusion Model (MKFM) that utilises knowledge produced by LLMs for Emotion Recognition in Conversations (ERC). This methodology incorporates co-reference resolution, topic extraction, and emotional cause identification, all produced by ChatGPT, to enhance the classification of emotions in conversational datasets. The research attained significant enhancements across three public datasets, illustrating the complementary nature of LLM-driven methodologies to sensor-based techniques in offering a comprehensive knowledge of emotional well-being. This study emphasises the capacity of LLMs to improve emotion recognition tasks by addressing the deficiency in annotated data and leveraging their contextual comprehension abilities.

The growing elderly population has heightened concerns about healthcare challenges associated with dementia, prompting research into emotion recognition using wearable devices. Zeng et al. [101] employed an LSTM network to analyze physiological signals such as EEG and ECG to distinguish emotional states in elderly individuals. The initial trials conducted using wearable textile devices demonstrated promising results, achieving a high accuracy of 95.00%. Nakisa et al. [102] advanced this area by utilizing affordable wearable sensors, including EEG and blood volume pressure (BVP), for emotion identification. They applied LSTM networks and introduced the Differential Evolution (DE) algorithm for LSTM hyperparameter optimization. This innovative approach resulted in a 14.00% improvement in accuracy, outperforming other methods highlighting the importance of optimization techniques in enhancing the performance of LSTM networks for emotion recognition using wearable sensors. Moreover, in the realm of socially assistive robotics, Tizzano et al. [104] explored the classification of mood using LSTM-based deep learning techniques. They integrated gyroscope, accelerometer, and heart rate measurements as inputs to the LSTM network, combined with Support Vector Machines (SVM). Their investigation achieved a correct prediction accuracy of up to 71.00%, demonstrating the challenges posed by individual variations in mood classification.

Lian et al. introduced a revolutionary wearable device designed with the capability to conduct a full analysis of the user's personal stress levels, as well as the emotions exhibited by individuals in their vicinity, and the environmental factors present in the surrounding area [106]. The system comprises several components, including a module for acquiring multi-dimensional physiological signals, a module for acquiring and transmitting images, a user interface on a mobile terminal, and a cloud database for storing data. In addition, the system incorporated a hybrid 2D-CNN-LSTM model that was evaluated using publicly accessible datasets successfully achieving 99.00% precision and recall. In a similar manner, scientists in [107] relied on multimodal data from various sensors, including physiological signals, environmental data, and motion data to perform emotion recognition by employing a CNN-LSTM model. The hybrid model achieved high accuracy (95.00%) without manual feature extraction. Another hybrid recurrent model was investigated in [108] with the utilisation of an EEG headset for the purpose of recognising emotions in the context of brain-computer interfaces (BCI). The proposed methodology employed a novel technique that transforms EEG data into two-dimensional matrices, enabling the collection of spatial correlation. Additionally, the study utilised a hybrid CNN-RNN model to categorise arousal and valence. The approach demonstrated a notable level of 93.00% classification accuracy, rendering it appropriate for use with consumer-grade multi-

electrode EEG headsets.

B. Self-esteem

Self-esteem can be defined as an individual's level of self-acceptance and self-regard, either at a particular moment or in a broader sense [88]. This evaluation is influenced by both societal and personal norms, as well as the individual's perception of their competence in areas of life that have significance to their personal and social identity [88]. Due to its inherently subjective nature, the assessment of self-esteem has primarily relied on self-report scales, with a lesser emphasis on implicit measures [123]. Self-evaluation is rooted in subjective, emotionally charged assessments of one's own identity. These evaluations might pertain to specific areas, such as professional achievements, athletic abilities, or physical attractiveness, as well as encompass a broader and more comprehensive assessment of one's overall self. A self that is distinct to a certain temporal period.

The assessment of self-esteem is a complex matter, as there are over 200 distinct scales that purportedly measure this construct [124]. Indicatively, the Single Item Self-Esteem scale (SISE) [125] and the Rosenberg Self-Esteem scale (RSE) [126] are used to measure the compenent of overall self-worth (i.e. overall attitude that ones holds about oneself). Furthermore, the State Self-Esteem Scale (SSES) measures the overall evaluation of the self [127], while the Multidimensional Self-Esteem Inventory (MSEI) is focused on assessing not only the global self-esteem but also the defensive self-esteem (i.e. defensive reaction of changes in one's own self-esteem) [128]. Lastly, the Contingency of Self-Worth scale (CSWs) measures seven domains of contingent self-worth [129].

Assessing or quantifying self-esteem through data collected from wearable devices is a complex and multifaceted challenge. While machine learning and data analysis can provide valuable insights into various aspects of a person's life, the notion that self-esteem is inherently subjective and not bound by objective criteria, arises several obstacles in assessing it directly from wearable device data. Nevertheless, in the presented review, we identified a study in which, authors introduced a novel approach that utilizes EEG data as input to a CNN model, achieving an accuracy rate of over 79.00% in distinguishing individuals with high and low self-esteem [109].

C. Spirituality

The term spirituality refers to a person exploration that entails acknowledging a sentiment or perception or conviction regarding the existence of a higher entity beyond oneself, a deeper aspect to human existence beyond sensory perception, and the understanding that the encompassing entirety in which we participate possesses cosmic or divine attributes [130].

Questionnaires can be utilised as a means of evaluating spirituality. Questionnaires are frequently employed in both academic and clinical contexts to assess a wide range of dimensions related to spirituality, beliefs, and experiences. The purpose of these evaluations is to measure individuals' cognitive, affective, and attitudinal dimensions of spirituality, offering a measurable approach to comprehending and researching this intricate facet of human existence. One of the commonly utilised tools for evaluating spirituality by researchers and clinicians is the Spiritual Well-Being Scale (SWBS [131]). This assessment instrument gauges an individual's perception of spiritual well-being through the examination of their sense of purpose, meaning in life, and connection to a higher power or broader reality. In addition, the Spiritual Needs Questionnaire (SpNQ) serves the purpose of evaluating and recording the spiritual challenges and/or requirements of patients, with the objective of facilitating the development of suitable strategies for support and planning [132]. Furthermore, the Spirituality Questionnaire assesses four distinct elements of spirituality, including belief in a higher power, quest for existential significance, state of mindfulness, and sense of emotional well-being [133].

The exploration of assessing spirituality in the context of QoL assessment through the utilisation of wearable sensors and DL (or machine learning) is undeniably an intriguing element to consider. Nevertheless, as previously stated, spirituality is a multifaceted and profoundly individualistic facet of an individual's existence, and quantifying it by technological means poses numerous obstacles. The primary focus of wearable sensors lies in the collection of objective, physical data, encompassing metrics such as heart rate, activity levels, and sleep patterns. The measurement of spirituality through sensors may need the utilisation of indirect indicators, which may not fully encapsulate the entirety of an individual's spiritual encounter. In particular, spiritual experiences frequently encompass mental and emotional states that may lack immediate physical manifestations that can be quantified by wearable technology. Furthermore, the experience of spirituality can be shaped by a multitude of contextual elements, including cultural, religious, and individual belief systems. DL models may encounter challenges in effectively capturing the intricacy and contextual nuances of the given task. In contrast to physical health criteria, the absence of a universally acknowledged ground truth or gold standard for assessing spirituality poses challenges in validating DL models.

D. Thinking

Thinking, also known as cognition, is a complex mental process that encompasses a wide range of activities and functions in the human mind. The process encompasses the acquisition, processing, storage, and utilisation of information in order to comprehend the world and navigate diverse facets of existence [134]. Cognition comprises a range of essential cognitive functions, including perception, attention, memory, problem-solving, decision-making, and language understanding. Essentially, it establishes the cognitive framework by which individuals see, interpret, and engage with their environment [134]. The significance of this phenomenon is evident in its impact on various aspects such as problem-solving, communication, learning, memory, emotional regulation, decision-making, independence, mental health, and the quality of relationships. The comprehension and cultivation of cognitive capacities play a crucial role in augmenting the general standard of living [135]

Our review identified an investigation that focused on utilizing functional connectivity measurements from EEG data in combination with DL techniques to categorize cognitive effort levels [110]. Multiple DL classifiers, including CNN, LSTM, and CNN-LSTM, were employed, with the combination of Mutual Information (MI) and CNN achieving the highest accuracy at 80.87%. Gasparini et al. investigated the widespread adoption of wearable sensors, specifically focusing on the utilisation of PPG technology, in order to assess blood volume and identify instances of heightened arousal resulting from stress and extreme cognitive load [111]. They demonstrated the efficacy of utilising a pretrained CNN to extract deep features for classification tasks by turning monodimensional PPG data into bidimensional signals. Their findings revealed that these deep features surpassed the performance of hand-crafted features in various classification tests reaching up to an accuracy of 79.00%. Moreover, a separate study introduced an end-to-end deep neural network using raw EEG signals for cognitive workload classification in vehicle drivers, showcasing its effectiveness in detecting cognitive burden [112]. The propsoed CNN model reached an average accuracy of 96.70% in classifying cognitive workload states.

In another study, a cost-effective EEG gadget designed for consumer usage is utilised to differentiate between states of focused concentration and relaxation [113] by employing a DNN model among other traditional ML approaches. The findings suggest that the development of a brain-machine interface using this particular device is viable, as it has demonstrated a notable level of accuracy of over 81.00%. Another DNN forward model was centred on the evaluation of cognitive burden levels through the utilisation of eye-tracking data [114]. The methodology of interpretable machine learning models was utilised to derive the metrics of feature importance and their impact on brain cognitive functions. The implemented approaches resulted in an enhancement of classification quality, while also reducing the number of utilised features to either six or eight, depending on the specific model. The primary evaluation criteria for classification performance, including F1 score, recall, precision, accuracy, and the area under the Receiver Operating Characteristic curve (ROC AUC), were employed to objectively analyse the classification quality. The highest achieved result on the whole set of features was 0.95 (f1-score). However, by interpreting the relevance of features, it was possible to improve the result to 0.97 using only seven out of the 20 features.

The investigation of attention monitoring using electroencephalography (EEG) in real-world scenarios was explored in [115]. In this investigation on the prediction of attention and distraction, the authors observed that the developed LSTM model achieved a classification accuracy of 95.86% and 95.40% for attention and distraction, respectively. Another approach involved recognizing human mental workload using DL principles and EEG features, utilizing a stacked denoising autoencoder (SDAE) with a feature mapping layer [116]. The ensemble SDAE classifier exhibited superior performance, outperforming traditional estimators with 92.02% accuracy.

E. Body Image

The concept of body image encompasses an individual's personal and subjective understanding, cognitive processes, emotional responses, and attitudes towards their own physical body, including factors such as its visual presentation, dimensions, form, and general physical characteristics [136]. This phenomenon covers both cognitive and emotional dimensions of an individual's self-perception, which can span from experiencing positive body pleasure to experiencing negative body dissatisfaction [137].

Currently, there is a wide range of smartwatches, activity trackers, and smart garments that can be found in the market that offer the potential to gather data on many bodily parameters such as blood pressure, body movements, and respiration [138]. These measurements are indicators often associated with specific body conditions than can be measured objectively such as obesity in children and adults [139], [140]. However, at present, we are still distant from the feasibility of offering comprehensive and dependable representations of individuals' bodies to users. These representations would serve to enhance their body awareness and provide them with an intelligent depiction of their physical form. Trackers primarily concentrate on "objective" metrics, and the way they provide information about the body is sometimes not intelligible for individuals who are not accustomed to handling extensive quantitative data. Furthermore, it should be noted that sensors are not specifically designed to capture several dimensions of body image, including body acceptance and love, the influence of inner optimism on outward demeanour, and the appreciation of the body's functions. To comprehensively consider all dimensions of the body image construct, it is insufficient to solely rely on the capabilities of existing trackers. It is imperative to incorporate self-reported metrics as well. Two of those are the Body-Image Acceptance and Action Questionnaire which measures the influence on binge eating [141] and the body image scale which is a simple and valid tool for assessing body image dissatisfaction in inflammatory bowel disease [142].

IV. Deep Learning and Quality of Life: A Comprehensive Discussion on Innovations, Challenges, and Future Prospects

In this review, an examination was conducted on the incorporation of DL methodologies in the evaluation of QoL, with particular emphasis on the subdomains of physical and psychological well-being, utilising data obtained from wearable devices. The present discussion section provides a more comprehensive examination of the ramifications, difficulties, and prospects linked to the use of deep learning in the assessment of QoL.

Section II, highlighted the adoption of DL techniques, particularly CNN, and LSTM from several studies that showcased remarkable accuracy in recognizing various daily activities, including driving behaviors, distracted driving, and human actions. Researchers are increasingly exploring also hybrid DL models that integrate different architectures to achieve higher accuracy and efficiency in HAR applications. Regarding the facet of Medication adherence, notable studies demonstrated

the effectiveness of DL in detecting medication administration motions using smartwatches, achieving high precision, and utilizing camera image sensors for medication consumption recognition. In the domain of chronic inflammatory pulmonary diseases, a deep sparse CNN was employed to assess medication adherence in real-time, reducing computational complexity while maintaining high accuracy. Additionally, studies on audio event identification and adherence detection among patients with type 2 diabetes (T2D) using DL methods showed significant potential in enhancing adherence monitoring. Moreover, we identified several studies that focused on detecting mental stress and fatigue using a variety of techniques. These include models like the Contractive Sparse Auto-encoder for identifying pilot fatigue with high accuracy, self-attention models and feature selection for fatigue assessment, and wearable devices for exercise exhaustion detection. In the context of road safety, researchers developed effective methods for driver drowsiness detection, incorporating factors like HRV and EEG signals to predict fatigue states with high accuracy.

Recent advancements in wearable technologies, such as EDA sensors, in combination with DL techniques, offered promising tools for pain measurement. Notable studies include the use of multi-task learning (MTL) neural networks for autonomous pain quantification using ECG inputs, achieving superior outcomes. Other approaches involved the assessment of pain in non-verbal individuals using either functional near-infrared spectroscopy (fNIRS) or EEG signals by using variable frequency band-based CNN models, and deep belief networks (DBN) in conjunction with PPG. Additionally, studies demonstrated the effectiveness of utilizing data from wearables for sleep quality assessment, with CNN achieving a high degree of accuracy. CNN also excelled in forecasting sleep quality from wearable data, outperforming other architectures. Notably, DL models were employed to automate the classification of sleep stages, with 1D-CNN and models like SleepEEGNet and DeepSleepNet achieving impressive accuracy in identifying different sleep stages. Actigraphy sensors proved also valuable for predicting sleep efficiency, with CNN demonstrating the highest accuracy.

In the domain of psychological well-being (Section III), the utilisation of wearable sensors has become indispensable for the instantaneous assessment of emotional conditions. Significant advancements in the field encompass the use of benchmark datasets such as eSEE-d for the purpose of approximating emotions through the application of wearable eye-tracking devices. These innovations have yielded commendable outcomes, particularly in terms of obtaining notable accuracy through the implementation of DNN. Furthermore, the application of wearable sensors, such as EEG and BVP, is commonly observed in the field of emotion identification. This is frequently achieved through the utilisation of LSTM networks and optimisation methods, such as Differential Evolution (DE). The application of LSTM-based DL techniques was employed in the field of socially assistive robotics to classify mood. This classification was achieved by utilising data from gyroscope, accelerometer, and HR sensors. Several previous studies utilised multimodal data and hybrid CNN-LSTM models to achieve a high level of accuracy in emotion recognition. These studies have even extended their application to brain-computer interfaces, showcasing the promise of wearable technology and deep learning in comprehending emotions and its influence on QoL.

Additionally, numerous studies investigated the utilisation of wearable technologies and DL algorithms in the evaluation of cognitive functioning. These methods included the analysis of EEG data using CNN, LSTM networks, and hybrid CNN-LSTM models. The primary objective of these analyses was to accurately classify different degrees of cognitive effort, which was successfully achieved. The integration of PPG technology and pretrained CNN models demonstrated efficacy in evaluating increased arousal resulting from stress and cognitive load, outperforming handmade features in terms of classification accuracy. Subsequent research endeavours have presented novel end-to-end DL architectures aimed at classifying cognitive workload. Notably, a CNN model was proposed for the purpose of accurately detecting cognitive burden in vehicle drivers, with remarkable results. Additional studies have been conducted in the realm of consumer-oriented EEG devices, with a specific emphasis on discerning states of focused attention and relaxation through the use of DNN models. These investigations demonstrated the practicality and potential of brain-machine interfaces.

Moreover, a comprehensive analysis was conducted on the merits and limitations of several datasets pertaining to the evaluation of QoL through the use of wearable data. The datasets encompass a broad spectrum of participant quantities and encompass a variety of age cohorts, hence enhancing the generalizability of the research findings. The distribution of gender exhibits variability across different datasets, so contributing to the comprehension of how demographic factors influence the assessment of QoL. The datasets included cover a diverse range of stimuli, ranging from everyday activities to sleep-related information, providing researchers with a broad range of options for conducting QoL studies. A range of wearable sensors, such as accelerometers, gyroscopes, ECG, EEG, and EMG, are utilised to provide comprehensive examination of QoL from diverse vantage points. In general, the wide range of open-access datasets offers researchers significant resources to investigate numerous aspects of QoL using wearable data, tailored to the individual requirements of distinct research inquiries.

Overall, DL methodology have brought about significant breakthroughs in the field of QoL measurement, surpassing traditional approaches like questionnaires. Conventional approaches, although useful, frequently depend on data provided by individuals themselves, which can be influenced by personal biases, memory mistakes, or a desire to conform to societal norms. In addition, these techniques can be time-consuming, require a lot of manual effort, and may not accurately capture immediate changes in QoL [143]. On the contrary, DL techniques bring about a fundamental change by utilising the capabilities of wearable technology and sophisticated algorithms to deliver unbiased, uninterrupted, and immediate observations on many aspects of quality of life. Based on the aforementioned results, the identified DL models showcased exceptional preci-

sion in identifying everyday activities, detecting adherence to medication, and evaluating mental states by utilising data from wearable devices [144]. These models not only streamline the process of collecting data but also analyse extensive amounts of data to reveal patterns, trends, and correlations that may not be immediately evident using conventional methods.

Furthermore, DL approaches demonstrated exceptional proficiency in managing multimodal data obtained from a variety of sensors such as ECG, EEG, and accelerometers. This capability enables a more comprehensive and nuanced comprehension of QoL, thus allowing researchers and healthcare providers to encompass a wider range of QoL indicators, including both physical health measurements and psychological well-being, in a more holistic manner [145]. In addition, DL algorithms possess the capability to adjust and develop alongside new data, delivering dynamic and personalised insights that can be customised to meet particular requirements. This flexibility and scalability is especially advantageous in healthcare environments because assessments of QoL must be responsive to variations over time and in diverse situations.

While this comprehensive review has made notable contributions by consolidating the most pertinent material on Deep Learning techniques for assessing QoL, it is crucial to recognise its inherent limits. A significant obstacle arises from the considerable disparity observed among research with regards to their data collection methods and the manner in which they communicate their findings. The presence of variability is a challenge in terms of conducting direct comparisons across the models employed in these investigations. Several research exhibited a lack of explicit documentation regarding the evaluation of model performance using a test set, and in some cases, the results from models may not have demonstrated optimal performance for the given study. Acceptability is a crucial concern, as patients and individuals may have hesitations about sharing personal data obtained through wearable devices. The apprehension over privacy, safeguarding of data, and the possible exploitation of confidential information can impact the inclination of individuals to engage in studies or utilise DL-based methods for assessing quality of life. Furthermore, the capacity of DL models to be applied effectively to different populations and environments continues to be a difficult task. Moreover, the majority of research concentrate on particular demographic groups or conditions, so restricting the generalizability of their findings to wider populations. The absence of generalizability can compromise the efficacy and pertinence of deep learning-based quality of life assessments in real-world healthcare environments. In addition, the examination of certain subdomains pertaining to physical and psychological well-being, such as spirituality and body image, using deep learning techniques presents several challenges. These facets exhibit intrinsic subjectivity and are not constrained by objective criteria.

Another factor to take into account is the ADL subdomain findings that are displayed in Table I. Despite the existence of numerous studies demonstrating a remarkably high level of classification accuracy, there are still notable obstacles that need to be addressed. First and foremost, it is challenging to apply these models to different populations because of the differences in demographic characteristics and activity patterns. Moreover, the performance of migrating models from controlled conditions to real-world applications is frequently diminished as a result of noise and unreliable sensor data. Effectively integrating several data sources and ensuring long-term monitoring with limited battery life of wearable devices are still persistent issues. Moreover, the flexibility of models to adjust to specific user behaviour and ensuring the confidentiality and protection of data are of utmost importance. As the complexity of models increases, it becomes more challenging to interpret and clarify the judgements made by these models. It is crucial to tackle these problems in order to make progress in human activity recognition and to guarantee the practical effectiveness of deep learning approaches in evaluating QoL.

Ultimately, while considering the future of assessing QoL using AI, there are various developing patterns that are influencing the overall scenario [146]. An important development is the incorporation of explainable AI approaches to enhance transparency and interpretability. Federated learning is increasingly becoming popular since it enables collaborative training of models while ensuring the privacy of data. The progress in edge computing allows for immediate analysis of data from wearable devices, hence increasing the accessibility of quality of life assessment. Customised deep learning models are now being created to adjust to individual quality of life metrics and preferences, hence improving their relevance and effectiveness. Interdisciplinary collaboration across neuroscience, psychology, and computer science is promoting creative research at the nexus of wearable technology, DL, and QoL evaluation. This collaboration is creating opportunities for significant breakthroughs in the field.

References

[1] "Who: Constitution of the World Health Organization."

[2] J. R. Turner, M. Wit, T. Hajos, M. Wit, M. B. Howren, S. Insana, and M. A. Simonson, "Quality of life," in *Encyclopedia of Behavioral Medicine* (M. D. Gellman and J. R. Turner, eds.), pp. 1602–1603, New York, NY: Springer New York, 2013.

[3] A. Alexandrova, *A philosophy for the science of well-being*, vol. 1. Oxford University Press, Aug. 2017.

[4] J. Addington-Hall and L. Kalra, "Who should measure quality of life?," *BMJ : British Medical Journal*, vol. 322, pp. 1417–1420, June 2001.

[5] H. M. Buiting and G. Olthuis, "Importance of quality-of-life measurement throughout the disease course," *JAMA Network Open*, vol. 3, p. e200388, Mar. 2020.

[6] M.-J. Linton, P. Dieppe, and A. Medina-Lara, "Review of 99 self-report measures for assessing well-being in adults: exploring dimensions of well-being and developments over time," *BMJ Open*, vol. 6, p. e010641, July 2016.

[7] N. E. Mayo, S. Figueiredo, S. Ahmed, and S. J. Bartlett, "Montreal Accord on Patient-Reported Outcomes (Pros) use series – Paper 2: terminology proposed to measure what matters in health," *Journal of Clinical Epidemiology*, vol. 89, pp. 119–124, Sept. 2017.

[8] I. B. Wilson, "Linking clinical variables with health-related quality of life: a conceptual model of patient outcomes," *JAMA*, vol. 273, p. 59, Jan. 1995.

[9] "Whoqol-bref| the world health organization."

[10] "Whoqol - measuring quality of life| the world health organization."

[11] K. Lekadir, A. Feragen, A. J. Fofanah, A. F. Frangi, A. Buyx, A. Emelie, A. Lara, A. R. Porras, A.-W. Chan, A. Navarro, B. Glocker, B. O. Botwe, B. Khanal, B. Beger, C. C. Wu, C. Cintas, C. P. Langlotz, D. Rueckert, D. Mzurikwao, D. I. Fotiadis, D. Zhussupov, E. Ferrante, E. Meijering, E. Weicken, F. A. González, F. W. Asselbergs, F. Prior, G. P. Krestin, G. Collins, G. S. Tegenaw, G. Kaissis, G. Misuraca,

G. Tsakou, G. Dwivedi, H. Kondylakis, H. Jayakody, H. C. Woodruf, H. J. Aerts, I. Walsh, I. Chouvarda, I. Buvat, I. Rekik, J. Duncan, J. Kalpathy-Cramer, J. Zahir, J. Park, J. Mongan, J. W. Gichoya, J. A. Schnabel, K. Kushibar, K. Riklund, K. Mori, K. Marias, L. M. Amugongo, L. A. Fromont, L. Maier-Hein, L. C. Alberich, L. Rittner, L. Phiri, L. Marrakchi-Kacem, L. Donoso-Bach, L. Martí-Bonmatí, M. J. Cardoso, M. Bobowicz, M. Shabani, M. Tsiknakis, M. A. Zuluaga, M. Bielikova, M.-C. Fritzsche, M. G. Linguraru, M. Wenzel, M. De Bruijne, M. G. Tolsgaard, M. Ghassemi, M. Ashrafuzzaman, M. Goisauf, M. Yaqub, M. Ammar, M. C. Abadía, M. M. E. Mahmoud, M. Elattar, N. Rieke, N. Papanikolaou, N. Lazrak, O. Díaz, O. Salvado, O. Pujol, O. Sall, P. Guevara, P. Gordebeke, P. Lambin, P. Brown, P. Abolmaesumi, Q. Dou, Q. Lu, R. Osuala, R. Nakasi, S. K. Zhou, S. Napel, S. Colantonio, S. Albarqouni, S. Joshi, S. Carter, S. Klein, S. E. Petersen, S. Aussó, S. Awate, T. R. Raviv, T. Cook, T. E. M. Mutsvangwa, W. A. Rogers, W. J. Niessen, X. Puig-Bosch, Y. Zeng, Y. G. Mohammed, Y. S. J. Aquino, Z. Salahuddin, and M. P. A. Starmans, "FUTURE-AI: International consensus guideline for trustworthy and deployable artificial intelligence in healthcare," Aug. 2023. arXiv:2309.12325 [cs].

[12] Z. Han, J. Zhao, H. Leung, K. F. Ma, and W. Wang, "A Review of Deep Learning Models for Time Series Prediction," *IEEE Sensors Journal*, vol. 21, pp. 7833–7848, Mar. 2021.

[13] N. Tsiknakis, E. Trivizakis, E. E. Vassalou, G. Z. Papadakis, D. A. Spandidos, A. Tsatsakis, J. Sánchez-García, R. López-González, N. Papanikolaou, A. H. Karantanas, and K. Marias, "Interpretable artificial intelligence framework for COVID-19 screening on chest X-rays," *Experimental and Therapeutic Medicine*, vol. 20, pp. 727–735, Aug. 2020.

[14] K. M. Tsiouris, A. Mitsis, G. Grigoriadis, G. Karanasiou, L. Lakkas, D. Mauri, M. A. Toli, A. Alexandraki, K. Keramida, D. Cardinale, and D. I. Fotiadis, "Risk stratification for cardiotoxicity in breast cancer patients: predicting early decline of lvef after treatment*," in *2023 45th Annual International Conference of the IEEE Engineering in Medicine & Biology Society (EMBC)*, pp. 1–4, July 2023. ISSN: 2694-0604.

[15] N. Tsiknakis, D. Theodoropoulos, G. Manikis, E. Ktistakis, O. Boutsora, A. Berto, F. Scarpa, A. Scarpa, D. I. Fotiadis, and K. Marias, "Deep learning for diabetic retinopathy detection and classification based on fundus images: A review," *Computers in Biology and Medicine*, vol. 135, p. 104599, Aug. 2021.

[16] D. Garg, G. K. Verma, and A. K. Singh, "A review of deep learning based methods for affect analysis using physiological signals," *Multimedia Tools and Applications*, vol. 82, pp. 26089–26134, July 2023.

[17] M. Beaudoin, A. Hudon, C.-E. Giguère, S. Potvin, and A. Dumais, "Prediction of quality of life in schizophrenia using machine learning models on data from Clinical Antipsychotic Trials of Intervention Effectiveness (Catie) schizophrenia trial," *Schizophrenia (Heidelberg, Germany)*, vol. 8, p. 29, Mar. 2022.

[18] P. K. Siogkas, D. S. Pleouras, V. D. Tsakanikas, V. T. Potsika, K. M. Tsiouris, A. Sakellarios, E. Karamouzi, F. Lagiou, G. Charalampopoulos, G. Galyfos, F. Sigala, I. Koncar, and D. I. Fotiadis, "A Machine Learning Model for the prediction of the progression of carotid arterial stenoses," in *2023 45th Annual International Conference of the IEEE Engineering in Medicine & Biology Society (EMBC)*, pp. 1–4, July 2023. ISSN: 2694-0604.

[19] V. Skaramagkas, A. Pentari, D. I. Fotiadis, and M. Tsiknakis, "Using the recurrence plots as indicators for the recognition of Parkinson's disease through phonemes assessment," in *2023 45th Annual International Conference of the IEEE Engineering in Medicine & Biology Society (EMBC)*, pp. 1–4, July 2023. ISSN: 2694-0604.

[20] R. Bommasani, D. A. Hudson, E. Adeli, R. Altman, S. Arora, S. von Arx, M. S. Bernstein, J. Bohg, A. Bosselut, E. Brunskill, E. Brynjolfsson, S. Buch, D. Card, R. Castellon, N. Chatterji, A. Chen, K. Creel, J. Q. Davis, D. Demszky, C. Donahue, M. Doumbouya, E. Durmus, S. Ermon, J. Etchemendy, K. Ethayarajh, L. Fei-Fei, C. Finn, T. Gale, L. Gillespie, K. Goel, N. Goodman, S. Grossman, N. Guha, T. Hashimoto, P. Henderson, J. Hewitt, D. E. Ho, J. Hong, K. Hsu, J. Huang, T. Icard, S. Jain, D. Jurafsky, P. Kalluri, S. Karamcheti, G. Keeling, F. Khani, O. Khattab, P. W. Koh, M. Krass, R. Krishna, R. Kuditipudi, A. Kumar, F. Ladhak, M. Lee, T. Lee, J. Leskovec, I. Levent, X. L. Li, X. Li, T. Ma, A. Malik, C. D. Manning, S. Mirchandani, E. Mitchell, Z. Munyikwa, S. Nair, A. Narayan, D. Narayanan, B. Newman, A. Nie, J. C. Niebles, H. Nilforoshan, J. Nyarko, G. Ogut, L. Orr, I. Papadimitriou, J. S. Park, C. Piech, E. Portelance, C. Potts, A. Raghunathan, R. Reich, H. Ren, F. Rong, Y. Roohani, C. Ruiz, J. Ryan, C. Ré, D. Sadigh, S. Sagawa, K. Santhanam, A. Shih, K. Srinivasan, A. Tamkin, R. Taori, A. W. Thomas, F. Tramèr, R. E. Wang, W. Wang, B. Wu, J. Wu, Y. Wu, S. M. Xie, M. Yasunaga, J. You, M. Zaharia, M. Zhang, T. Zhang, X. Zhang, Y. Zhang, L. Zheng, K. Zhou, and P. Liang, "On the opportunities and risks of foundation models," July 2022. arXiv:2108.07258 [cs].

[21] S. M. A. Iqbal, I. Mahgoub, E. Du, M. A. Leavitt, and W. Asghar, "Advances in healthcare wearable devices," *npj Flexible Electronics*, vol. 5, pp. 1–14, Apr. 2021.

[22] V. Skaramagkas, A. Pentari, Z. Kefalopoulou, and M. Tsiknakis, "Multi-modal deep learning diagnosis of parkinson's disease—a systematic review," *IEEE Transactions on Neural Systems and Rehabilitation Engineering*, vol. 31, pp. 2399–2423, 2023.

[23] M. Xu, L. Guo, and H.-C. Wu, "Novel Robust Automatic Brain-Tumor Detection and Segmentation Using Magnetic Resonance Imaging," *IEEE Sensors Journal*, vol. 24, pp. 10957–10964, Apr. 2024.

[24] E. Mbunge, B. Muchemwa, S. Jiyane, and J. Batani, "Sensors and healthcare 5.0: transformative shift in virtual care through emerging digital health technologies," *Global Health Journal*, vol. 5, pp. 169–177, Dec. 2021.

[25] Y. Xue, "A review on intelligent wearables: Uses and risks," *Human Behavior and Emerging Technologies*, vol. 1, pp. 287–294, Oct. 2019.

[26] K. El-Raheb, V. Kalampratsidou, P. Issari, E. Georgaca, F. Koliouli, E. Karydi, T. D. Skali, P. Diamantides, and Y. Ioannidis, "Wearables in sociodrama: An embodied mixed-methods study of expressiveness in social interactions," *Wearable Technologies*, vol. 3, p. e10, Jan. 2022.

[27] CARDIOCARE CONSORTIUM, "AN INTERDISCIPLINARY APPROACH FOR THE MANAGEMENT OF THE ELDERLY MULTIMORBID PATIENT WITH BREAST CANCER THERAPY INDUCED CARDIAC TOXICITY," *CORDIS | European Commission*, Jan. 2021.

[28] G. Karanasiou, L. Koumakis, S. Sfakianakis, G. Manikis, G. Kalliatakis, A. Antoniades, L. Lakkas, D. Mauri, C. Cipolla, K. Mazzocco, A. Papakonstantinou, G. Filippatos, A. Constantinidou, B. Šeruga, C. Conti, A. Bucur, E. Pacella, K. Marias, M. Tsiknakis, and D. Fotiadis, "CARDIOCARE: An integrated platform for the management of elderly multimorbid patients with breast cancer therapy induced cardiac toxicity," in *2023 45th Annual International Conference of the IEEE Engineering in Medicine & Biology Society (EMBC)*, pp. 1–4, July 2023. ISSN: 2694-0604.

[29] H. N. Rasmussen, M. F. Scheier, and J. B. Greenhouse, "Optimism and physical health: a meta-analytic review," *Annals of Behavioral Medicine*, vol. 37, pp. 239–256, June 2009.

[30] N. Dua, S. N. Singh, and V. B. Semwal, "Multi-input cnn-gru based human activity recognition using wearable sensors," *Computing*, vol. 103, pp. 1461–1478, 7 2021.

[31] R. Zhu, Z. Xiao, Y. Li, M. Yang, Y. Tan, L. Zhou, S. Lin, and H. Wen, "Efficient human activity recognition solving the confusing activities via deep ensemble learning," *IEEE Access*, vol. 7, pp. 75490–75499, 2019.

[32] A. Mohamed, F. Lejarza, S. Cahail, C. Claudel, and E. Thomaz, "Hargcnn: deep graph cnns for human activity recognition from highly unlabeled mobile sensor data," in *2022 IEEE International Conference on Pervasive Computing and Communications Workshops and other Affiliated Events (PerCom Workshops)*, pp. 335–340, Mar. 2022. arXiv:2203.03087 [cs].

[33] Q. Teng, K. Wang, L. Zhang, and J. He, "The layer-wise training convolutional neural networks using local loss for sensor-based human activity recognition," *IEEE Sensors Journal*, vol. 20, pp. 7265–7274, July 2020.

[34] Y. Xing, C. Lv, H. Wang, D. Cao, E. Velenis, and F.-Y. Wang, "Driver activity recognition for intelligent vehicles: a deep learning approach," *IEEE Transactions on Vehicular Technology*, vol. 68, pp. 5379–5390, June 2019.

[35] F. Kuncan, Y. Kaya, Z. Yiner, and M. Kaya, "A new approach for physical human activity recognition from sensor signals based on motif patterns and long-short term memory," *Biomedical Signal Processing and Control*, vol. 78, 9 2022.

[36] A. Sarkar, S. K. S. Hossain, and R. Sarkar, "Human activity recognition from sensor data using spatial attention-aided CNN with genetic algorithm," *Neural Computing and Applications*, vol. 35, pp. 5165–5191, Mar. 2023.

[37] I. Dirgová Luptáková, M. Kubovčík, and J. Pospíchal, "Wearable sensor-based human activity recognition with transformer model," *Sensors*, vol. 22, p. 1911, Jan. 2022.

[38] S. Mekruksavanich, P. Jantawong, N. Hnoohom, and A. Jitpattanakul, "Refined lstm network for sensor-based human activity recognition in real world scenario," in *2022 IEEE 13th International Conference on*

Software Engineering and Service Science (ICSESS), pp. 256–259, Oct. 2022. ISSN: 2327-0594.

[39] K. Xia, J. Huang, and H. Wang, "Lstm-cnn architecture for human activity recognition," *IEEE Access*, vol. 8, pp. 56855–56866, 2020.

[40] S. K. Challa, A. Kumar, and V. B. Semwal, "A multibranch cnn-bilstm model for human activity recognition using wearable sensor data," *Visual Computer*, vol. 38, pp. 4095–4109, 12 2022.

[41] P. Agarwal and M. Alam, "A lightweight deep learning model for human activity recognition on edge devices," *Procedia Computer Science*, vol. 167, pp. 2364–2373, 1 2020.

[42] R. Mutegeki and D. S. Han, "A cnn-lstm approach to human activity recognition," in *2020 International Conference on Artificial Intelligence in Information and Communication (ICAIIC)*, pp. 362–366, Feb. 2020.

[43] V. B. Semwal, A. Gupta, and P. Lalwani, "An optimized hybrid deep learning model using ensemble learning approach for human walking activities recognition," *Journal of Supercomputing*, vol. 77, pp. 12256–12279, 11 2021.

[44] C. O. Odhiambo, L. Ablonczy, P. J. Wright, C. F. Corbett, S. Reichardt, and H. Valafar, "Detecting medication-taking gestures using machine learning and accelerometer data collected via smartwatch technology: instrument validation study," *JMIR human factors*, vol. 10, p. e42714, May 2023.

[45] H. Lee and S. Youm, "Development of a wearable camera and ai algorithm for medication behavior recognition," *Sensors (Basel, Switzerland)*, vol. 21, p. 3594, May 2021.

[46] V. Ntalianis, S. Nousias, A. S. Lalos, M. Birbas, N. Tsafas, and K. Moustakas, "Assessment of medication adherence in respiratory diseases through deep sparse convolutional coding," in *2019 24th IEEE International Conference on Emerging Technologies and Factory Automation (ETFA)*, pp. 1657–1660, Sept. 2019. ISSN: 1946-0759.

[47] D. Pettas, S. Nousias, E. I. Zacharaki, and K. Moustakas, "Recognition of breathing activity and medication adherence using lstm neural networks," in *2019 IEEE 19th International Conference on Bioinformatics and Bioengineering (BIBE)*, pp. 941–946, Oct. 2019. ISSN: 2471-7819.

[48] D. N. Thyde, A. Mohebbi, H. Bengtsson, M. L. Jensen, and M. Mørup, "Machine learning-based adherence detection of type 2 diabetes patients on once-daily basal insulin injections," *Journal of Diabetes Science and Technology*, vol. 15, pp. 98–108, Jan. 2021.

[49] E. Q. wu, P.-Y. Deng, X.-Y. Qiu, Z. Tang, W.-M. Zhang, L.-M. Zhu, H. Ren, G.-R. Zhou, and R. S. F. Sheng, "Detecting fatigue status of pilots based on deep learning network using eeg signals," *IEEE Transactions on Cognitive and Developmental Systems*, vol. 13, pp. 575–585, Sept. 2021.

[50] Y. Bai, Y. Guan, and W.-F. Ng, "Fatigue assessment using ecg and actigraphy sensors," in *Proceedings of the 2020 International Symposium on Wearable Computers*, pp. 12–16, Sept. 2020. arXiv:2008.02871 [cs, stat].

[51] X. Guan, Y. Lin, Q. Wang, Z. Liu, and C. Liu, "Sports fatigue detection based on deep learning," in *2021 14th International Congress on Image and Signal Processing, BioMedical Engineering and Informatics (CISP-BMEI)*, pp. 1–6, Oct. 2021.

[52] Y. Wang, Y. Huang, B. Gu, S. Cao, and D. Fang, "Identifying mental fatigue of construction workers using EEG and deep learning," *Automation in Construction*, vol. 151, p. 104887, July 2023.

[53] U. Budak, V. Bajaj, Y. Akbulut, O. Atila, and A. Sengur, "An effective hybrid model for eeg-based drowsiness detection," *IEEE Sensors Journal*, vol. 19, pp. 7624–7631, Sept. 2019.

[54] C. Wang, T. S. Kumar, W. De Raedt, G. Camps, H. Hallez, and B. Vanrumste, "Eating speed measurement using wrist-worn imu sensors in free-living environments," Dec. 2023. arXiv:2401.05376 [cs, eess].

[55] D. Utomo, T.-H. Yang, D. T. Thanh, and P.-A. Hsiung, "Driver fatigue prediction using different sensor data with deep learning," in *2019 IEEE International Conference on Industrial Cyber Physical Systems (ICPS)*, pp. 242–247, May 2019.

[56] V. P. Balam, V. U. Sameer, and S. Chinara, "Automated classification system for drowsiness detection using convolutional neural network and electroencephalogram," *IET Intelligent Transport Systems*, vol. 15, pp. 514–524, Apr. 2021.

[57] S. Sharma and A. Hoover, "Top-down detection of eating episodes by analyzing large windows of wrist motion using a convolutional neural network," *Bioengineering*, vol. 9, p. 70, Feb. 2022.

[58] H. Lee, J. Lee, and M. Shin, "Using wearable ecg/ppg sensors for driver drowsiness detection based on distinguishable pattern of recurrence plots," *Electronics*, vol. 8, p. 192, Feb. 2019.

[59] P. Kulurkar, C. k. Dixit, V. C. Bharathi, A. Monikavishnuvarthini, A. Dhakne, and P. Preethi, "AI based elderly fall prediction system using wearable sensors: A smart home-care technology with IOT," *Measurement: Sensors*, vol. 25, p. 100614, Feb. 2023.

[60] P.-K. Yang, B. Filtjens, P. Ginis, M. Goris, A. Nieuwboer, M. Gilat, P. Slaets, and B. Vanrumste, "Freezing of gait assessment with inertial measurement units and deep learning: effect of tasks, medication states, and stops," *Journal of NeuroEngineering and Rehabilitation*, vol. 21, p. 24, Feb. 2024.

[61] W. T. Mo and J. H. Chan, "Freezing of gait prediction using deep learning," in *Proceedings of the 13th International Conference on Advances in Information Technology*, IAIT '23, (New York, NY, USA), pp. 1–6, Association for Computing Machinery, Dec. 2023.

[62] S. Gkikas and M. Tsiknakis, "Automatic assessment of pain based on deep learning methods: A systematic review," *Computer Methods and Programs in Biomedicine*, vol. 231, p. 107365, Apr. 2023.

[63] R. F. Rojas, J. Romero, J. Lopez-Aparicio, and K. L. Ou, "Pain assessment based on fnirs using bi-lstm rnns," *International IEEE/EMBS Conference on Neural Engineering, NER*, vol. 2021-May, pp. 399–402, 5 2021.

[64] F. Pouromran, Y. Lin, and S. Kamarthi, "Personalized deep bi-lstm rnn based model for pain intensity classification using eda signal," *Sensors 2022, Vol. 22, Page 8087*, vol. 22, p. 8087, 10 2022.

[65] B. Hu, C. Kim, X. Ning, and X. Xu, "Using a deep learning network to recognise low back pain in static standing," *https://doi.org/10.1080/00140139.2018.1481230*, vol. 61, pp. 1374–1381, 10 2018.

[66] C. Wang, T. A. Olugbade, A. Mathur, A. C. Williams, N. D. Lane, and N. Bianchi-Berthouze, "Chronic pain protective behavior detection with deep learning," *ACM Transactions on Computing for Healthcare*, vol. 2, p. 23, 7 2021.

[67] J. Wang, M. Wei, L. Zhang, G. Huang, Z. Liang, L. Li, and Z. Zhang, "An autoencoder-based approach to predict subjective pain perception from high-density evoked eeg potentials," *Proceedings of the Annual International Conference of the IEEE Engineering in Medicine and Biology Society, EMBS*, vol. 2020-July, pp. 1507–1511, 7 2020.

[68] M. Yu, Y. Sun, B. Zhu, L. Zhu, Y. Lin, X. Tang, Y. Guo, G. Sun, and M. Dong, "Diverse frequency band-based convolutional neural networks for tonic cold pain assessment using eeg," *Neurocomputing*, vol. 378, pp. 270–282, 2 2020.

[69] H. Lim, B. Kim, G. J. Noh, and S. K. Yoo, "A deep neural network-based pain classifier using a photoplethysmography signal," *Sensors 2019, Vol. 19, Page 384*, vol. 19, p. 384, 1 2019.

[70] J. O. Pinzon-Arenas, Y. Kong, K. H. Chon, and H. F. Posada-Quintero, "Design and evaluation of deep learning models for continuous acute pain detection based on phasic electrodermal activity," *IEEE Journal of Biomedical and Health Informatics*, 2023.

[71] O. Kilic, B. Saylam, and O. D. Incel, "Sleep quality prediction from wearables using convolution neural networks and ensemble learning," pp. 116–120, 3 2023.

[72] A. Arora, P. Chakraborty, and M. P. Bhatia, "Analysis of data from wearable sensors for sleep quality estimation and prediction using deep learning," *Arabian Journal for Science and Engineering*, vol. 45, pp. 10793–10812, 12 2020.

[73] O. Yildirim, U. B. Baloglu, and U. R. Acharya, "A deep learning model for automated sleep stages classification using psg signals," *International Journal of Environmental Research and Public Health*, vol. 16, 2 2019.

[74] S. Mousavi, F. Afghah, and U. R. Acharya, "Sleepeegnet: Automated sleep stage scoring with sequence to sequence deep learning approach," *PLOS ONE*, vol. 14, p. e0216456, 5 2019.

[75] A. Supratak, H. Dong, C. Wu, and Y. Guo, "Deepsleepnet: a model for automatic sleep stage scoring based on raw single-channel eeg," *IEEE Transactions on Neural Systems and Rehabilitation Engineering*, vol. 25, pp. 1998–2008, 3 2017.

[76] A. Sathyanarayana, S. Joty, L. Fernandez-Luque, F. Ofli, J. Srivastava, A. Elmagarmid, T. Arora, and S. Taheri, "Sleep quality prediction from wearable data using deep learning," *JMIR mHealth and uHealth*, vol. 4, 10 2016.

[77] D. V. Phan, C. L. Chan, and D. K. Nguyen, "Applying deep learning for prediction sleep quality from wearable data," *ACM International Conference Proceeding Series*, pp. 51–55, 8 2020.

[78] S. Matsumori, K. Teramoto, H. Iyori, T. Soda, S. Yoshimoto, and H. Mizutani, "Haru sleep: A deep learning-based sleep scoring system with wearable sheet-type frontal eeg sensors," *IEEE Access*, vol. 10, pp. 13624–13632, 2022.

[79] M. Z. Uddin and A. Soylu, "Human activity recognition using wearable sensors, discriminant analysis, and long short-term memory-based

neural structured learning," *Scientific Reports*, vol. 11, p. 16455, Aug. 2021.

[80] L. Osterberg and T. Blaschke, "Adherence to medication," *The New England Journal of Medicine*, vol. 353, pp. 487–497, Aug. 2005.

[81] P. Kardas, P. Lewek, and M. Matyjaszczyk, "Determinants of patient adherence: a review of systematic reviews," *Frontiers in Pharmacology*, vol. 4, 2013.

[82] B. D. Loy, M. H. Cameron, and P. J. O'Connor, "Perceived fatigue and energy are independent unipolar states: supporting evidence," *Medical hypotheses*, vol. 113, pp. 46–51, Apr. 2018.

[83] W. W. H. Organization., *International classification of functioning, disability and health(Icf)*. 2nd ed. Geneva, 2001.

[84] J. Phillips, E. Dal Grande, C. Ritchie, A. P. Abernethy, and D. C. Currow, "A population-based cross-sectional study that defined normative population data for the Life-Space Mobility Assessment-composite score," *Journal of Pain and Symptom Management*, vol. 49, pp. 885–893, May 2015.

[85] A. Kuspinar, C. P. Verschoor, M. K. Beauchamp, J. Dushoff, J. Ma, E. Amster, C. Bassim, V. Dal Bello-Haas, M. A. Gregory, J. E. Harris, L. Letts, S. E. Neil-Sztramko, J. Richardson, R. Valaitis, and B. Vrkljan, "Modifiable factors related to life-space mobility in community-dwelling older adults: results from the Canadian Longitudinal Study on Aging," *BMC geriatrics*, vol. 20, p. 35, Jan. 2020.

[86] D. Thakur, A. Guzzo, and G. Fortino, "Attention-based multihead deep learning framework for online activity monitoring with smartwatch sensors," *IEEE Internet of Things Journal*, pp. 1–1, 2023.

[87] V. D. Tsakanikas, D. G. Dimopoulos, N. S. Tachos, C. Chatzaki, V. Skaramagkas, G. Christodoulakis, M. Tsiknakis, and D. I. Fotiadis, "Gait and balance patterns related to Free-Walking and TUG tests in Parkinson's Disease based on plantar pressure data," in *2021 43rd Annual International Conference of the IEEE Engineering in Medicine & Biology Society (EMBC)*, pp. 236–239, Nov. 2021. ISSN: 2694-0604.

[88] K. Wac and S. Wulfovich, eds., *Quantifying quality of life: incorporating daily life into medicine*. Health Informatics, Cham: Springer International Publishing, 2022.

[89] S. Gkikas, C. Chatzaki, and M. Tsiknakis, "Multi-task neural networks for pain intensity estimation using electrocardiogram and demographic factors," *Communications in Computer and Information Science*, vol. 1856 CCIS, pp. 324–337, 2023.

[90] D. Kocevska, T. S. Lysen, A. Dotinga, M. E. Koopman-Verhoeff, M. P. C. M. Luijk, N. Antypa, N. R. Biermasz, A. Blokstra, J. Brug, W. J. Burk, H. C. Comijs, E. Corpeleijn, H. S. Dashti, E. J. de Bruin, R. de Graaf, I. P. M. Derks, J. F. Dewald-Kaufmann, P. J. M. Elders, R. J. B. J. Gemke, L. Grievink, L. Hale, C. A. Hartman, C. J. Heijnen, M. Huisman, A. Huss, M. A. Ikram, S. E. Jones, M. K. Velderman, M. Koning, A. M. Meijer, K. Meijer, R. Noordam, A. J. Oldehinkel, J. O. Groeniger, B. W. J. H. Penninx, H. S. J. Picavet, S. Pieters, S. A. Reijneveld, E. Reitz, C. M. Renders, G. Rodenburg, F. Rutters, M. C. Smith, A. S. Singh, M. B. Snijder, K. Stronks, M. ten Have, J. W. R. Twisk, D. Van de Mheen, J. van der Ende, K. B. van der Heijden, P. G. van der Velden, F. J. van Lenthe, R. R. L. van Litsenburg, S. H. van Oostrom, F. J. van Schalkwijk, C. M. Sheehan, R. A. Verheij, F. C. Verhulst, M. C. M. Vermeulen, R. C. H. Vermeulen, W. M. M. Verschuren, T. G. M. Vrijkotte, A. H. Wijga, A. M. Willemen, M. ter Wolbeek, A. R. Wood, Y. Xerxa, W. M. Bramer, O. H. Franco, A. I. Luik, E. J. W. Van Someren, and H. Tiemeier, "Sleep characteristics across the lifespan in 1.1 million people from the Netherlands, United Kingdom and United States: a systematic review and meta-analysis," *Nature Human Behaviour*, vol. 5, pp. 113–122, Jan. 2021.

[91] D. Riebe, J. Ehrman, G. Liguori, and M. Magal, *ACSM's Guidelines for Exercise Testing and Prescription*. 03 2018.

[92] R. S. Falck, S. McDonald, M. Beets, K. Brazendale, and T. Liu-Ambrose, "Physical activity measurement in older adult interventions: a systematic review and meta-analysis: 897 board #293 may 27, 3: 30 pm - 5: 00 pm," *Medicine & Science in Sports & Exercise*, vol. 47, p. 247, May 2015.

[93] L. Turner-Stokes, J. Fadyl, H. Rose, H. Williams, P. Schlüter, and K. McPherson, "The work-ability support scale: evaluation of scoring accuracy and rater reliability," *Journal of Occupational Rehabilitation*, vol. 24, no. 3, pp. 511–524, 2014.

[94] S. J. Isernhagen, "Functional capacity evaluation: Rationale, procedure, utility of the kinesiophysical approach," *Journal of Occupational Rehabilitation*, vol. 2, pp. 157–168, Sept. 1992.

[95] J. Ilmarinen, "The work ability index(Wai)," *Occupational Medicine*, vol. 57, pp. 160–160, Oct. 2006.

[96] "Work capacity test information," Dec. 2017.

[97] P. Salovey, A. J. Rothman, J. B. Detweiler, and W. T. Steward, "Emotional states and physical health.," *American Psychologist*, vol. 55, pp. 110–121, Jan. 2000.

[98] V. Skaramagkas, E. Ktistakis, D. Manousos, E. Kazantzaki, N. S. Tachos, E. Tripoliti, D. I. Fotiadis, and M. Tsiknakis, "esee-d: Emotional state estimation based on eye-tracking dataset," *Brain Sciences 2023, Vol. 13, Page 589*, vol. 13, p. 589, 3 2023.

[99] Z. Wang, Z. Yu, B. Zhao, B. Guo, C. Chen, and Z. Yu, "Emotionsense," *ACM Transactions on Computing for Healthcare*, vol. 1, p. 2020, 9 2020.

[100] V. Dissanayake, S. Seneviratne, R. Rana, E. Wen, T. Kaluarachchi, and S. Nanayakkara, "Sigrep: Toward robust wearable emotion recognition with contrastive representation learning," *IEEE Access*, vol. 10, pp. 18105–18120, 2022.

[101] F. Zeng, Y. Lin, P. Siriaraya, D. Choi, and N. Kuwahara, "Emotion detection using eeg and ecg signals from wearable textile devices for elderly people," *Journal of Textile Engineering*, vol. 66, pp. 109–117, 12 2020.

[102] B. Nakisa, M. N. Rastgoo, A. Rakotonirainy, F. Maire, and V. Chandran, "Long short term memory hyperparameter optimization for a neural network based emotion recognition framework," *IEEE Access*, vol. 6, pp. 49325–49338, 8 2018.

[103] G. Tu, B. Liang, B. Qin, K.-F. Wong, and R. Xu, "An empirical study on multiple knowledge from chatgpt for emotion recognition in conversations," in *Findings of the Association for Computational Linguistics: EMNLP 2023* (H. Bouamor, J. Pino, and K. Bali, eds.), (Singapore), pp. 12160–12173, Association for Computational Linguistics, Dec. 2023.

[104] G. R. Tizzano, M. Spezialetti, and S. Rossi, "A deep learning approach for mood recognition from wearable data," *IEEE Medical Measurements and Applications, MeMeA 2020 - Conference Proceedings*, 6 2020.

[105] J. C. Quiroz, E. Geangu, and M. H. Yong, "Emotion recognition using smart watch sensor data: mixed-design study," *JMIR Mental Health*, vol. 5, p. e10153, Aug. 2018.

[106] Z. Lian, Y. Guo, X. Cao, and W. Li, "An ear wearable device system for facial emotion recognition disorders," *Frontiers in Bioengineering and Biotechnology*, vol. 9, p. 703048, 6 2021.

[107] E. Kanjo, E. M. Younis, and C. S. Ang, "Deep learning analysis of mobile physiological, environmental and location sensor data for emotion detection," *Information Fusion*, vol. 49, pp. 46–56, 9 2019.

[108] J. Chen, D. Jiang, Y. Zhang, and P. Zhang, "Emotion recognition from spatiotemporal eeg representations with hybrid convolutional recurrent neural networks via wearable multi-channel headset," *Computer Communications*, vol. 154, pp. 58–65, 3 2020.

[109] R. Buettner, D. Sauter, I. Eckert, and H. Baumgartl, "Classifying high and low self-esteem using a novel machine learning method based on eeg data," *PACIS 2021 Proceedings*, 7 2021.

[110] A. Gupta, G. Siddhad, V. Pandey, P. P. Roy, and B.-G. Kim, "Subject-specific cognitive workload classification using eeg-based functional connectivity and deep learning," *Sensors*, vol. 21, p. 6710, Jan. 2021.

[111] F. Gasparini, A. Grossi, and S. Bandini, "A deep learning approach to recognize cognitive load using ppg signals," in *Proceedings of the 14th PErvasive Technologies Related to Assistive Environments Conference*, PETRA '21, (New York, NY, USA), pp. 489–495, Association for Computing Machinery, June 2021.

[112] M. A. Almogbel, A. H. Dang, and W. Kameyama, "Cognitive workload detection from raw eeg-signals of vehicle driver using deep learning," in *2019 21st International Conference on Advanced Communication Technology (ICACT)*, pp. 1–6, 2019.

[113] S. D. You, "Classification of relaxation and concentration mental states with eeg," *Information*, vol. 12, p. 187, May 2021.

[114] M. Kaczorowska, M. Plechawska-Wójcik, and M. Tokovarov, "Interpretable machine learning models for three-way classification of cognitive workload levels for eye-tracking features," *Brain Sciences*, vol. 11, p. 210, Feb. 2021.

[115] P. Kaushik, A. Moye, M. v. Vugt, and P. P. Roy, "Decoding the cognitive states of attention and distraction in a real-life setting using EEG," *Scientific Reports*, vol. 12, p. 20649, Nov. 2022.

[116] S. Yang, Z. Yin, Y. Wang, W. Zhang, Y. Wang, and J. Zhang, "Assessing cognitive mental workload via EEG signals and an ensemble deep learning classifier based on denoising autoencoders," *Computers in Biology and Medicine*, vol. 109, pp. 159–170, June 2019.

[117] Z. Yin and J. Zhang, "Cross-session classification of mental workload levels using EEG and an adaptive deep learning model," *Biomedical Signal Processing and Control*, vol. 33, pp. 30–47, Mar. 2017.

[118] A. Damasio, "Fundamental feelings," *Nature*, vol. 413, pp. 781–781, Oct. 2001.

[119] J.-C. Chebat and R. Michon, "Impact of ambient odors on mall shoppers' emotions, cognition, and spending: A test of competitive causal theories," *Journal of Business Research*, vol. 56, pp. 529–539, July 2003.

[120] V. Skaramagkas, G. Giannakakis, E. Ktistakis, D. Manousos, I. Karatzanis, N. Tachos, E. Tripoliti, K. Marias, D. I. Fotiadis, and M. Tsiknakis, "Review of eye tracking metrics involved in emotional and cognitive processes," *IEEE reviews in biomedical engineering*, vol. 16, pp. 260–277, 2023.

[121] S. Saganowski, B. Perz, A. Polak, and P. Kazienko, "Emotion recognition for everyday life using physiological signals from wearables: A systematic literature review," *IEEE Transactions on Affective Computing*, 2022.

[122] J. Hayano, T. Tanabiki, S. Iwata, K. Abe, and E. Yuda, "Estimation of emotions by wearable biometric sensors under daily activities," *2018 IEEE 7th Global Conference on Consumer Electronics, GCCE 2018*, pp. 268–269, 12 2018.

[123] T. F. Heatherton and C. L. Wyland, "Assessing self-esteem.," in *Positive psychological assessment: A handbook of models and measures.* (S. J. Lopez and C. R. Snyder, eds.), pp. 219–233, Washington: American Psychological Association, 2003.

[124] T. J. Scheff and D. S. Fearon, "Cognition and emotion? The dead end in self-esteem research," *Journal for the Theory of Social Behaviour*, vol. 34, pp. 73–90, Mar. 2004.

[125] R. W. Robins, H. M. Hendin, and K. H. Trzesniewski, "Measuring global self-esteem: construct validation of a single-item measure and the rosenberg self-esteem scale," *Personality and Social Psychology Bulletin*, vol. 27, pp. 151–161, Feb. 2001.

[126] M. Rosenberg, "Society and the adolescent self-image," in *Society and the Adolescent Self-Image*, Princeton University Press, Dec. 2015.

[127] T. F. Heatherton and J. Polivy, "Development and validation of a scale for measuring state self-esteem.," *Journal of Personality and Social Psychology*, vol. 60, pp. 895–910, June 1991.

[128] R. W. Tafarodi and W. B. Swann, "Two-dimensional self-esteem: theory and measurement," *Personality and Individual Differences*, vol. 31, pp. 653–673, Oct. 2001.

[129] J. Crocker and C. T. Wolfe, "Contingencies of self-worth.," *Psychological Review*, vol. 108, pp. 593–623, July 2001.

[130] M. A. Burkhardt, "Spirituality: An analysis of the concept," *Holistic Nursing Practice*, vol. 3, p. 69, May 1989.

[131] R. Gomez and J. W. Fisher, "Domains of spiritual well-being and development and validation of the Spiritual Well-Being Questionnaire," *Personality and Individual Differences*, vol. 35, pp. 1975–1991, Dec. 2003.

[132] A. Büssing, "The spiritual needs questionnaire in research and clinical application: a summary of findings," *Journal of Religion and Health*, vol. 60, pp. 3732–3748, Oct. 2021.

[133] J. Hardt, S. Schultz, C. Xander, G. Becker, and M. Dragan, "The spirituality questionnaire: core dimensions of spirituality," *Psychology*, vol. 03, no. 01, pp. 116–122, 2012.

[134] M. C. A. B. Anderson, Michael W. Eysenck, *Memory*. London: Psychology Press, 2 ed., Nov. 2014.

[135] E. Ktistakis, V. Skaramagkas, D. Manousos, N. S. Tachos, E. Tripoliti, D. I. Fotiadis, and M. Tsiknakis, "COLET: A dataset for COgnitive workLoad estimation based on eye-tracking," *Computer Methods and Programs in Biomedicine*, vol. 224, p. 106989, Sept. 2022.

[136] C. Baker and E. H. Wertheim, "Body image: a handbook of theory, research, and clinical practice, edited by thomas f. Cash and thomas pruzinsky, new york: guilford press, 2002, 530 pages, $60. 00.," *Eating Disorders*, vol. 11, pp. 247–248, Sept. 2003.

[137] T. L. Tylka and N. L. Wood-Barcalow, "What is and what is not positive body image? Conceptual foundations and construct definition," *Body Image*, vol. 14, pp. 118–129, June 2015.

[138] E. Mencarini, A. Rapp, L. Tirabeni, and M. Zancanaro, "Designing wearable systems for sports: A review of trends and opportunities in human–computer interaction," *IEEE Transactions on Human-Machine Systems*, vol. 49, no. 4, pp. 314–325, 2019.

[139] M. Gupta, T. L. T. Phan, H. T. Bunnell, and R. Beheshti, "Obesity prediction with ehr data: A deep learning approach with interpretable elements," *ACM Transactions on Computing for Healthcare*, vol. 3, 4 2022.

[140] S. A. Thamrin, D. S. Arsyad, H. Kuswanto, A. Lawi, and S. Nasir, "Predicting obesity in adults using machine learning techniques: An analysis of indonesian basic health research 2018," *Frontiers in Nutrition*, vol. 8, p. 669155, 6 2021.

[141] P. Lucena-Santos, S. A. Carvalho, M. d. S. Oliveira, and J. Pinto-Gouveia, "Body-Image Acceptance and Action Questionnaire: Its deleterious influence on binge eating and psychometric validation," *International Journal of Clinical and Health Psychology*, vol. 17, pp. 151–160, May 2017.

[142] E. McDermott, J. Moloney, N. Rafter, D. Keegan, K. Byrne, G. A. Doherty, G. Cullen, K. Malone, and H. E. Mulcahy, "The body image scale: a simple and valid tool for assessing body image dissatisfaction in inflammatory bowel disease," *Inflammatory Bowel Diseases*, vol. 20, pp. 286–290, Feb. 2014.

[143] A. Jankowska, K. Młyńczak, and D. Golicki, "Validity of EQ-5D-5L health-related quality of life questionnaire in self-reported diabetes: evidence from a general population survey," *Health and Quality of Life Outcomes*, vol. 19, p. 138, Dec. 2021.

[144] S. F. Ahmed, M. S. B. Alam, M. Hassan, M. R. Rozbu, T. Ishtiak, N. Rafa, M. Mofijur, A. B. M. Shawkat Ali, and A. H. Gandomi, "Deep learning modelling techniques: current progress, applications, advantages, and challenges," *Artificial Intelligence Review*, vol. 56, pp. 13521–13617, Nov. 2023.

[145] S. K. Sahu, A. Mokhade, and N. D. Bokde, "An overview of machine learning, deep learning, and reinforcement learning-based techniques in quantitative finance: recent progress and challenges," *Applied Sciences*, vol. 13, p. 1956, Jan. 2023.

[146] A. T. G. Tapeh and M. Z. Naser, "Artificial intelligence, machine learning, and deep learning in structural engineering: a scientometrics review of trends and best practices," *Archives of Computational Methods in Engineering*, vol. 30, pp. 115–159, Jan. 2023.
